# Rab29-dependent asymmetrical activation of leucine-rich repeat kinase 2

**DOI:** 10.1126/science.adi9926

**Published:** 2023-12-21

**Authors:** Hanwen Zhu, Francesca Tonelli, Martin Turk, Alan Prescott, Dario R. Alessi, Ji Sun

**Affiliations:** 1 Department of Structural Biology, St. Jude Children’s Research Hospital, Memphis, TN 38105, USA; 2 MRC Protein Phosphorylation and Ubiquitylation Unit, School of Life Sciences, University of Dundee, Dundee DD1 5EH, UK; 3 Aligning Science Across Parkinson’s (ASAP) Collaborative Research Network, Chevy Chase, MD, USA; 4 Cryo-EM and Tomography Center, St. Jude Children’s Research Hospital, Memphis, TN 38105, USA

## Abstract

Gain-of-function mutations in *LRRK2*, which encodes the leucine-rich repeat kinase 2 (LRRK2), are the most common genetic cause of late-onset Parkinson’s Disease. LRRK2 is recruited to membrane organelles and activated by Rab29, a Rab guanosine triphosphatase encoded in the *PARK16* locus. We present cryo–electron microscopy structures of Rab29–LRRK2 complexes in three oligomeric states, providing key snapshots during LRRK2 recruitment and activation. Rab29 induces an unexpected tetrameric assembly of LRRK2, formed by two kinase-active central protomers and two kinase-inactive peripheral protomers. The central protomers resemble the active-like state trapped by the type I kinase inhibitor DNL201, a compound that underwent a phase 1 clinical trial. Our work reveals the structural mechanism of LRRK2 spatial regulation and provides insights into LRRK2 inhibitor design for Parkinson’s disease treatment.

Parkinson’s disease (PD) is the second most prevalent neurodegenerative disorder, affecting 1 to 2% of the population over the age of 65 (1). Mutations in the *LRRK2* gene, which encodes the leucine-rich repeat kinase 2 (LRRK2) protein, are among the most frequent genetic causes of late-onset PD and account for ~5% of familial and ~1% of sporadic cases (2–4). More than 250 mutations in *LRRK2* have been identified, and ~100 were biochemically characterized (5–7). Most LRRK2-related PD mutations, such as G2019S (Gly^2019^→Ser), have increased kinase activity. Therefore, LRRK2 inhibitors are of great interest to researchers in PD treatment (2).

Much effort has been directed at exploring the structure-function relationship in LRRK2 to gain a mechanistic understanding and to guide rational drug discovery (8). Previous work revealed high-resolution structures of inactive LRRK2 (9, 10), whereas our understanding of LRRK2 activation is limited to microtubule-associated LRRK2 structures of disease mutations at mediate and low resolutions (11, 12) or molecular dynamics (MD) simulations in combination with hydrogen-deuterium exchange mass spectrometry (HDX-MS) analyses (13–16). Furthermore, LRRK2 activation in both physiological and pathogenic conditions is tightly associated with membrane recruitment and Rab guanosine triphosphatases (GTPases) (17–19), the molecular basis of which remains enigmatic.

This study aimed to explore the kinase activation mechanism of LRRK2 that is induced by Rab29, a membrane-anchored Rab GTPase. Rab GTPases are master regulators of intracellular-vesicle trafficking, whose disruption is a hallmark of PD pathogenesis (20). Rab29, encoded in the *PARK16* locus and associated with late-onset PD, is believed to function in the same pathway as LRRK2. Although mouse genetic data suggest that alternative activation mechanisms of LRRK2 exist (21), physiological connections between Rab29 and LRRK2 are supported by human and *Caenorhabditis elegans* genetic data, similar mouse knockout histological phenotypes, cellular colocalization, and physical interactions (22–27). Moreover, Rab29, but not its close homologs Rab32 or Rab38, stimulates LRRK2 kinase activity by monitoring S1292 autophosphorylation (28), suggesting that Rab29 does more than membrane recruitment to stimulate LRRK2 activity.

## Structural determination of Rab29–LRRK2 complexes

**Fig. 1.**
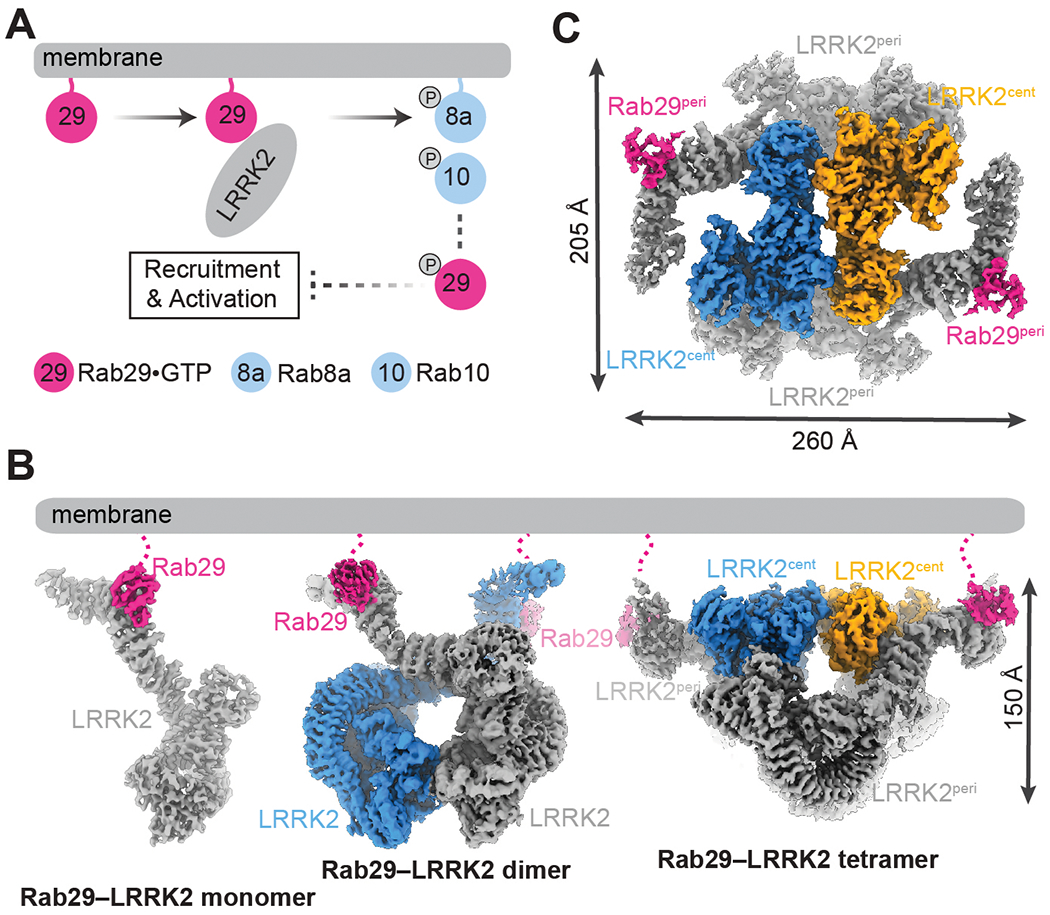
Structural determination of the Rab29–LRRK2 complex. (**A**) Schematic diagram showing Rab29-mediated LRRK2 membrane recruitment and activation. (**B**) Cryo-EM structures of the Rab29–LRRK2 complex in three oligomerization states. (**C**) Top view of the cryo-EM structure of the Rab29–LRRK2 tetramer.

To structurally characterize the Rab29–LRRK2 interaction, we first reconstituted a stable complex in vitro. Previous studies have suggested an emerging “Rab29–LRRK2–Rabs” cascade for LRRK2 signaling (Fig. 1A) (19, 29), in which GTP-bound Rab29 (Rab29•GTP) facilitates LRRK2 membrane recruitment and activation, then activated LRRK2 phosphorylates Rab GTPases, including Rab29 itself. Phosphorylated Rab29, which was reported not to activate LRRK2, could potentially serve as negative feedback (28). We thus introduced three point mutations (Q67L, T71A, and S72A) to Rab29 (Rab29_EM_) to maximize the opportunity of capturing the active Rab29•GTP–LRRK2 complex (where Q is glutamine, L is leucine, T is threonine, A is alanine, and S is serine). T71A and S72A prevent Rab29 phosphorylation, and Q67L abolishes GTPase activity and enhances the interaction between Rab29 and LRRK2 (26). Although Q67L also diminishes the membrane localization of Rab29 in cells (26), we reasoned that the interaction between Rab29 and LRRK2 is independent of membrane environment or composition (30) and should not be affected in vitro by this mutation. Indeed, glutathione *S*-transferase (GST) pulldown assays validated the Rab29_EM_–LRRK2 interaction and complex formation (fig. S2G).

We determined cryo–electron microscopy (cryo-EM) structures of the Rab29–LRRK2 complex, reconstituted by mixing LRRK2, Rab29_EM_, GTP analog (GppNHp), adenosine triphosphate (ATP), and Mg^2+^. The final cryo-EM reconstruction resulted in Rab29–LRRK2 structures of three distinct oligomeric assemblies (Fig. 1, B and C; fig. S1; and table S1): Rab29–LRRK2 monomer with one LRRK2 and one Rab29; Rab29–LRRK2 dimer with two LRRK2 and two Rab29; and Rab29–LRRK2 tetramer with four LRRK2 and two visible Rab29 (Fig. 1, B and C). Focused three-dimensional (3D) refinement improved the cryo-EM density surrounding the Rab29–LRRK2 interface (fig. S1A), and C2 symmetry was imposed for Rab29–LRRK2 dimer and tetramer during data analysis (fig. S1A).

## Rab29-dependent recruitment of LRRK2

**Fig. 2.**
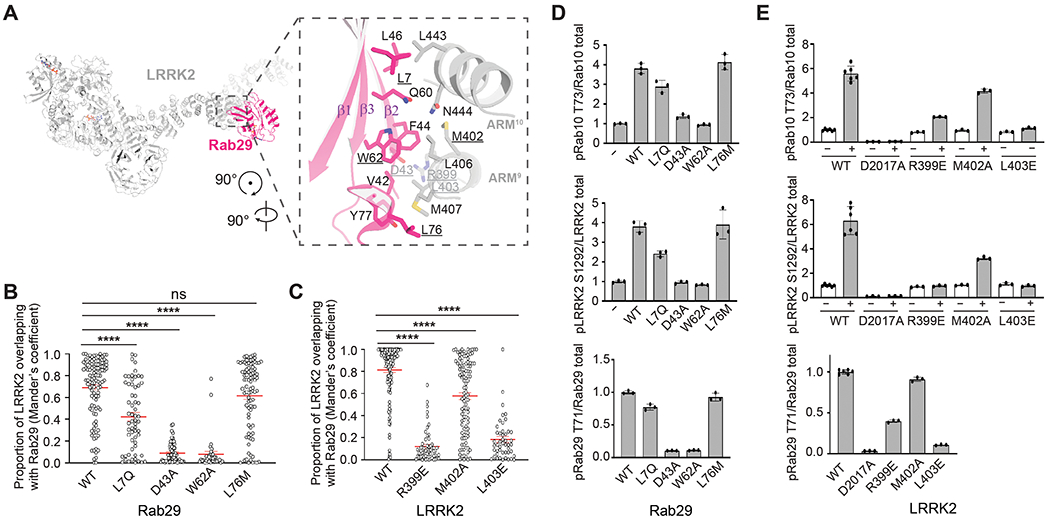
Molecular basis of Rab29-dependent LRRK2 recruitment. (**A**) Rab29–LRRK2 interface in the LRRK2 monomer state. LRRK2 and Rab29 are colored gray and hot pink, respectively. Side chains of interface residues are shown as sticks. (**B** and **C**) Impact of Rab29 or LRRK2 mutations on LRRK2 localization in HEK293 cells. Quantification of a portion of LRRK2 overlapping with Rab29 according to Mander’s coefficient for confocal analysis is shown in fig. S3, A and B. Each empty circle represents colocalization coefficient (Mander’s coefficient) measured in one cell. Error bars represent SEM. Significance was determined by the Kruskal-Wallis one-way analysis of variance (ANOVA) test. **** *P* < 0.0001; ns (not significant). (**D** and **E**) Quantification of the immunoblotting data shown in fig. S3, C and D. Data are presented as ratios of pRab10-Thr^73^/total Rab10, pLRRK2-Ser^1292^/total LRRK2, and pRab29-Thr^71^/total Rab29, normalized to the average of LRRK2 WT values. The data shown are the mean ± SD of three determinations. Single-letter abbreviations for the amino acid residues are as follows: A, Ala; C, Cys; D, Asp; E, Glu; F, Phe; G, Gly; H, His; I, Ile; K, Lys; L, Leu; M, Met; N, Asn; P, Pro; Q, Gln; R, Arg; S, Ser; T, Thr; V, Val; W, Trp; and Y, Tyr.

In the Rab29–LRRK2 monomer, LRRK2 is almost identical to the inactive LRRK2-alone structure (10) (fig. S2A). Rab29 binds to the N-terminal armadillo repeat (ARM) domain of LRRK2, burying a surface area of ~800 Å^2^ (Fig. 2A), and adopts a GTP-bound Switch I closed configuration often observed in GTP-bound small GTPases (fig. S2, B to E). Docking of the GDP-bound Rab29 structure (25) into the cryo-EM density shows obvious steric clashes with LRRK2 (fig. S2F), explaining why the GTP-bound state promotes Rab29-dependent recruitment of LRRK2 (24, 25, 27, 28, 30, 31).

The Rab29–LRRK2 interface is formed by the ARM9-10 of LRRK2 and the Switch I-Interswitch-Switch II surface and CDR1 (32) of Rab29 (fig. S2E). The interaction conforms with a general Rab-effector recognition mode, in which effectors associate with the GTP-bound form of Rabs through the Switch I-Interswitch-Switch II surface (32). Sequence alignment of Rab GTPases, including the Rab32 subfamily (Rab29, Rab32, and Rab38) and several LRRK2 substrates, revealed that key residues in the Rab29–LRRK2 interface were conserved among the Rab32 subfamily (fig. S2H). Single mutations at the center of the interface (Rab29 D43A or W62A) were sufficient to abolish the interaction, whereas Rab29 L7Q or L76M substitutions at the edge of the interface had moderate or little impact (fig. S2G). The surfaces of Rab29 and Rab10 predicted to interact with LRRK2 had almost identical interface residues, which is consistent with previous work suggesting that Rab10 interacts with LRRK2 at the Rab29 site (33). Rab5A, 5B, and 5C, which can be phosphorylated by LRRK2 (34), contain an alanine in the position corresponding to Asp^43^ in Rab29 (fig. S2H).

Consistent with the pulldown-assay results, confocal microscopy revealed that Rab29 D43A or W62A mutations diminished the membrane recruitment of LRRK2, whereas Rab29 L7Q or L76M substitutions had moderate or little disruptive effects (Fig. 2B and fig. S3A). Similarly, LRRK2 mutations (R399E and L403E) at the center of the Rab29–LRRK2 interface significantly reduced the Rab29-dependent membrane recruitment, whereas a mutation at the periphery of the interface (M402A) had a moderate impact (Fig. 2C and fig. S3B). We also assessed the effect of interface mutations on LRRK2 kinase activity in cells by monitoring the phosphorylation status of Rab10-Thr^73^, Rab29-Thr^71^, and LRRK2-Ser^1292^ (28). In agreement with the pulldown and membrane-localization results, mutations at the center of the interface on either the Rab29 (Fig. 2D and fig. S3, C and E) or LRRK2 side (Fig. 2E and fig. S3D) abolished the Rab29-stimulated kinase activity, whereas mutations at the periphery had only a minor impact. These data confirmed the observed Rab29–LRRK2 interface and suggested the importance of membrane recruitment for LRRK2 activation. All mutations introduced to the Rab29–LRRK2 interface had minor impacts on the basal activity of LRRK2 on Rab10 and Ser^1292^, supporting the view that the observed interface is important for LRRK2 recruitment but probably not for substrate recognition.

In the Rab29–LRRK2 dimer assembly, each LRRK2 protomer binds a single Rab29 molecule at the ARM9-10 interface (Fig. 1B). In this X-shaped complex, LRRK2 protomers adopt the same inactive conformation observed in the Rab29–LRRK2 monomer and the LRRK2-alone structure (fig. S4) (10). The two LRRK2 protomers interact via their COR-B domains, in a way that is similar to what we described for the LRRK2 homodimer (fig. S4B) (10). The observation of LRRK2 dimers both in the presence and absence of Rab29 suggests that COR-B–mediated dimerization of LRRK2 could occur under physiological settings.

## A tetrameric assembly of LRRK2

**Fig. 3.**
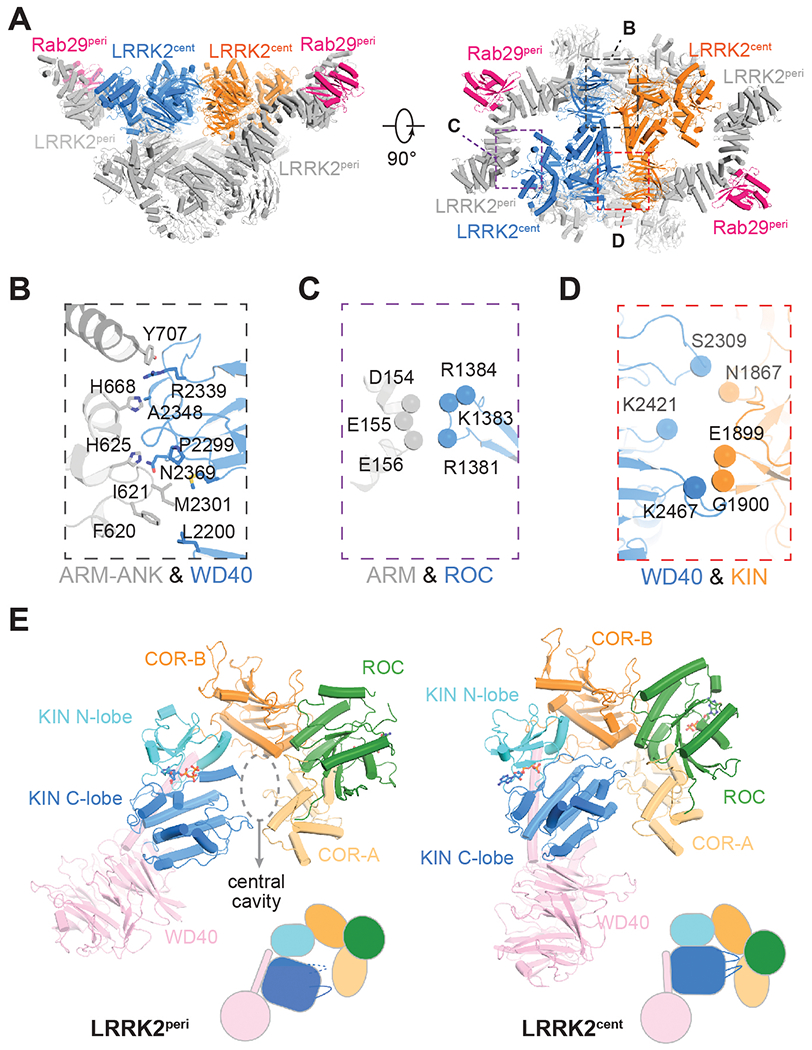
Structure of the Rab29–LRRK2 tetramer. (**A**) Cryo-EM structure of the Rab29–LRRK2 tetramer with two different views. Peripheral Rab29 (Rab29^peri^) and LRRK2 (LRRK2^peri^) are colored in hot pink and gray, respectively; central LRRK2 (LRRK2^cent^) copies are colored in blue and orange. (**B** and **C**) Interactions between (B) the WD40 domain of LRRK2^cent^ and ARM-ANK domains of LRRK2^peri^ and (C) the ROC domain of LRRK2^cen^ and the ARM domain of LRRK2^peri^. (**D**) Interactions between two LRRK2^cent^ copies. (**E**) Conformational changes in the C-terminal halves of LRRK2 upon activation. A dashed circle indicates the central cavity between the KIN and COR domains. Color codes for different parts of LRRK2 are as follows: ROC, green; COR-A, light orange; COR-B, bright orange; N-lobe of KIN, cyan; C-lobe of KIN, marine; WD40, pink.

We captured the Rab29–LRRK2 complex in an unexpected tetrameric assembly and determined its structure to an overall resolution of 3.5 Å (fig. S1 and table S1). With a ~205 Å by 260 Å by 150 Å dimension (Fig. 1, B and C), the Rab29–LRRK2 tetramer is an assembly with a twofold rather than fourfold symmetry, featuring two types of LRRK2 protomers: LRRK2^peri^ (peripheral) and LRRK2^cent^ (central) (Fig. 1, B and C, and Fig. 3A). We were able to resolve and model near full-length LRRK2^peri^ and associated Rab29^peri^. By contrast, LRRK2^cent^ protomers have flexible LRR, ankyrin repeat (ANK), and ARM domains, and neither those domains nor the associated Rab29^cent^ molecules could be resolved (Fig. 3A and fig. S5, A to C). By contrast, the catalytic halves of LRRK2^cent^, including ROC, COR, KIN, and WD40 domains (ROC, Ras of complex proteins; COR, C-terminal of ROC; KIN, kinase), were rigid and could be refined to an overall resolution of 3.2 Å with focused refinement (fig. S1A).

The LRRK2^peri^–LRRK2^cent^ interaction within each asymmetric unit is mediated by the COR-B domains (fig. S5, C and D), and the COR-B–COR-B interface is similar to that seen in LRRK2 homodimers or Rab29–LRRK2 dimers, with a subtle rotational motion (fig. S5D). In addition, LRRK2^peri^ directly interacts with LRRK2^cent^ from the other asymmetric unit (Fig. 3, A to C), with regions near the LRRK2^peri^ ARM–ANK boundary packing against the LRRK2^cent^ WD40 domain and the LRRK2^peri^ ARM domain associated with the LRRK2^cent^ ROC domain. The two LRRK2^cent^ protomers pack in a “head-to-tail” mode through WD40–KIN interfaces (Fig. 3, A and D). The flexible N-terminal part of LRRK2^cent^ might also interact with the Rab29^peri^ and the ARM domain of LRRK2^peri^, but the low local resolution of the cryo-EM map prevents further interpretation (fig. S5A).

The catalytic halves of LRRK2^cent^ and LRRK2^peri^ show substantial conformational differences (Fig. 3E and movie S1). Upon aligning the COR-B domain, KIN and WD40 are displaced about 40° toward the COR-A and ROC domains in LRRK2^cent^ (fig. S5E). This conformational rearrangement closes a central cavity shaped by the ROC, COR, and KIN domains (Fig. 3E). In this conformation, COR-B, ROC-COR-A, KIN N-lobe, and KIN C-lobe-WD40 appear to move as rigid bodies (fig. S5F). Repositioning of KIN C-lobe-WD40 in LRRK2^cent^ disrupts the connection between the WD40 and ARM-ANK-LRR domains in the inactive state, which was stabilized by the scaffolding hinge helix and C-terminal helix (fig. S5, G and H) (10). Additionally, the KIN C-lobe would clash into the LRR domain (fig. S5I), contributing to the displacement and flexibility of the ARM-ANK-LRR domains in LRRK2^cent^ protomers (fig. S5A).

## LRRK2^cent^ has an active kinase domain

**Fig. 4.**
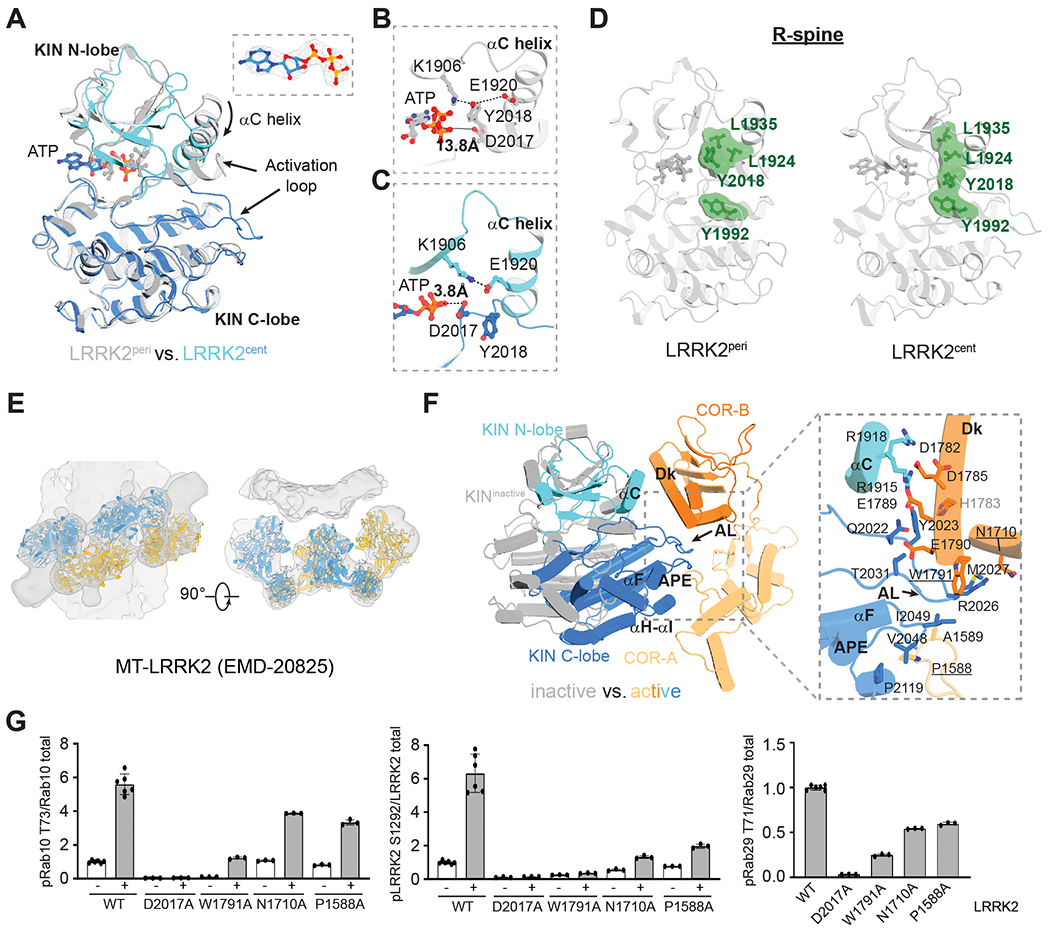
An active conformation of LRRK2. (**A**) Superposition of kinase domains of LRRK2^cent^ and LRRK2^peri^. N- and C-lobes of the LRRK2^cent^ kinase domain are colored in cyan and marine, respectively; the LRRK2^peri^ KIN domain is colored in gray. (Inset) Image shows the Cryo-EM density of the ATP molecule. (**B** and **C**) Key catalytic residues in LRRK2^peri^ (B) and LRRK2^cent^ (C) KIN domain with side chains shown as ball-and-stick models. The distances between the side chain of D2017 and the phosphate group of ATP are indicated with dashed lines. (**D**) R-spine of the LRRK2^peri^ (left) and LRRK2^cent^ (right) KIN domains. The four residues forming the R-spine (L1935, L1924, Y2018, and Y1992) are shown as green surfaces. (**E**) Docking of C-terminal catalytic halves of LRRK2^cent^ into the cryo-ET map of microtubule-bound LRRK2. (**F**) Movement of the KIN domain relative to the COR domain upon activation. (Inset) Interactions between the KIN and COR domains in the active conformation; side chains of the interface residues are shown as sticks. Dk, docking helix; APE, conserved APE motif; AL, activation loop. (**G**) Quantitative immunoblotting analysis of the cellular kinase activity of LRRK2-bearing mutations in the interface between the KIN and COR domains in fig. S6C. Data are presented as ratios of pRab10-Thr^73^/total Rab10, pLRRK2-Ser^1292^/total LRRK2, and pRab29-Thr^71^/total Rab29, normalized to the average of LRRK2 WT values. The data shown are the mean ± SD of three experiments.

The KIN domain of LRRK2^cent^ has structural features of an active kinase. The LRR domain that shields the KIN domain in the inactive conformation (10) is flexible in LRRK2^cent^, leaving the KIN domain accessible to substrates from the membrane side (fig. S5, A and B). Critically, the LRRK2^cent^ KIN domain adopts a closed conformation (Fig. 4A), with the αC helix positioned toward the active site and the “DYG motif’ flipped in. Lys^1906^ and Glu^1920^ form a salt bridge, an interaction blocked by Tyr^2018^ in the inactive conformation (Fig. 4, B and C). There is a well-defined cryo-EM density for ATP in the active site, and the distance between Asp^2017^ and ATP shortens to 3.8 Å (from 13.8 Å in the inactive state) (Fig. 4, A and C), permitting ATP hydrolysis in the presence of substrates. The regulatory spine (R-spine), formed by Leu^1935^, Leu^1924^, Tyr^2018^, and Tyr^1992^ becomes continuous (Fig. 4D). Docking of the LRRK2^cent^ model into the 14-Å in situ cryo–electron tomography (cryo-ET) map of microtubule-bound LRRK2 (Fig. 4E), which was proposed to represent an active conformation (11), reveals close correspondence, supporting our conclusion that the LRRK2^cent^ KIN domain is in an active conformation.

We next examined the interdomain interactions that stabilize the active conformation of the LRRK2^cent^ KIN domain. The activation loop of the KIN domain (35) dips into the open pocket between COR-A and COR-B (Fig. 4F). We hypothesized that this interdomain interaction stabilizes the closed conformation of the KIN domain and would thus be crucial for LRRK2 kinase activity. Indeed, single point mutations at the KIN–COR interface (P1588A, N1710A, and W1791A) reduced the LRRK2 kinase activity induced by Rab29 (Fig. 4G and fig. S6). LRRK2-W1791A almost completely abolished LRRK2 activity in the absence of Rab29, indicating that the observed interactions (Fig. 4F) are also critical for the basal activity of LRRK2 (Fig. 4G and fig. S6C). Therefore, blocking the COR–KIN interaction could be a potential strategy to inhibit LRRK2 allosterically.

Compared with the inactive state, the KIN N-lobe rotates slightly toward the COR-B domain in LRRK2^cent^, leading to more-extensive interactions between the αC helix of the KIN domain and the docking (Dk) helix of the COR-B domain (Fig. 4F). These observations are consistent with previous HDX-MS and MD simulation studies that indicated an altered interface between the COR-B Dk helix and the KIN αC helix and the stabilization of a nearby COR-B loop (residues 1721 to 1725) upon binding of type I inhibitors (fig. S5J) (15).

The ROC domain is displaced relative to the COR-B domain upon LRRK2 activation (fig. S5K). COR-B structurally bridges the catalytic ROC and KIN domains, and GTP binding in the former modulates the kinase activity of the latter (36–39). The movement of the ROC domain relative to the COR-B domain upon activation involves a “seesaw-like” motion of the ROC αC helix, with Tyr^1699^ as the pivot point (fig. S5K and movie S2). Our structural observations indicate that conformational coupling between the ROC and COR-B domains is vital for LRRK2 activity by contributing to the crosstalk between GTPase and kinase activities (10).

**Fig. 5.**
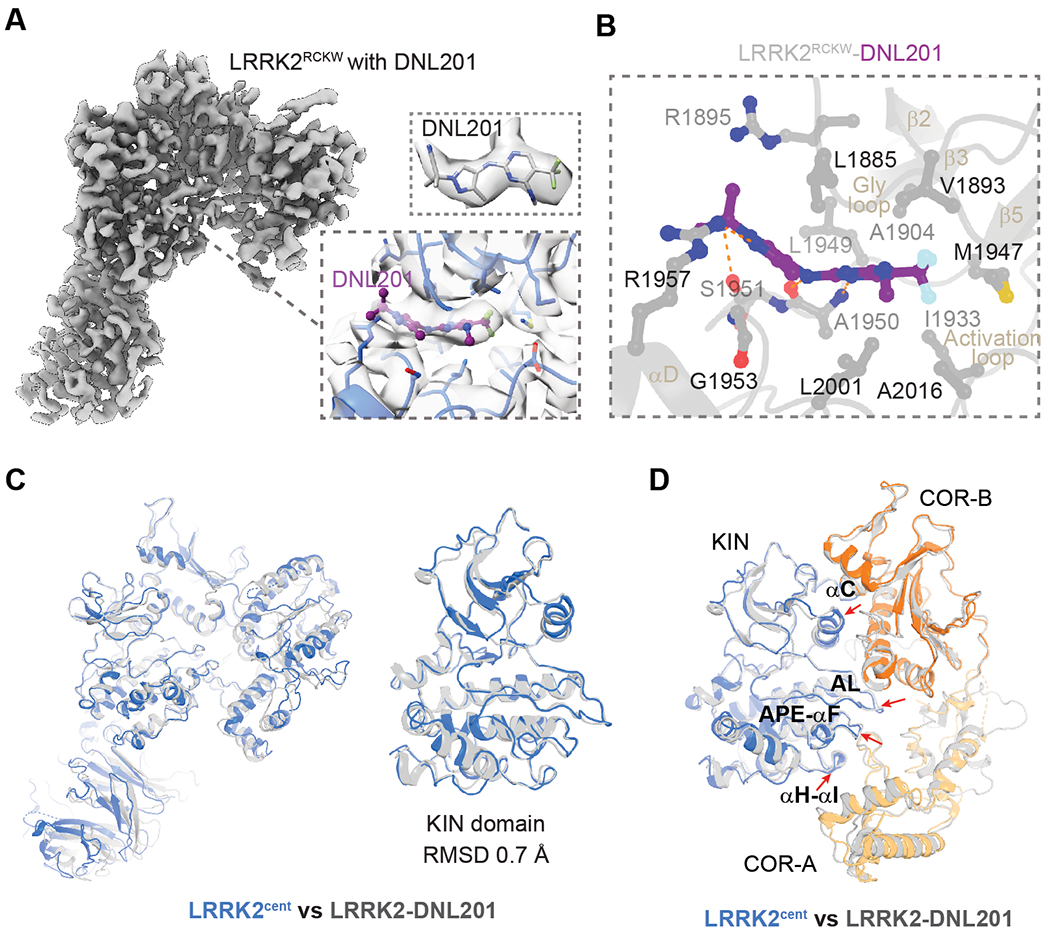
Structure of LRRK2^RCKW^ with DNL201. (**A**) Cryo-EM map of LRRK2^RCKW^ in complex with type I inhibitor DNL201. (Insets) Cryo-EM densities of the DNL201 inhibitor (top) and the surrounding residues (bottom) are shown. (**B**) DNL201 binding site [magnified from bottom inset of (A)]. Side chains of DNL201-interacting residues are shown as sticks. (**C**) Structural comparison of DNL201-bound LRRK2^RCKW^ and LRRK2^cent^ structures. (**D**) Comparison of KIN-ROC interface between LRRK2^RCKW^-DNL201 (gray) and LRRK2^cent^ (blue and orange). Key structural elements from KIN domain involved in the interaction are labeled.

We then determined the cryo-EM structure of LRRK2^RCKW^ (RCKW: ROC-COR-KIN-WD40) in complex with DNL201/GNE-0877, a compound reported to be safe and well tolerated in a phase 1 clinical trial (40) (Fig. 5A and fig. S7, A to H). LRRK2^RCKW^ is used to simplify the structure determination caused by the flexibility of the N-terminal domains. DNL201 is a type I kinase inhibitor that fixes the LRRK2 kinase domain in an active-like conformation, as judged by the compound’s ability to induce dephosphorylation of Ser^935^ (40, 41). The well-resolved kinase domain structure revealed a binding site for DNL201 within LRRK2 at the ATP-binding pocket (Fig. 5B and fig. S7, F to H). In the LRRK2^cent^ and LRRK2^RCKW^–DNL201 structures, KIN domains adopted a highly similar structure [root mean square deviation (RMSD), 0.7 Å] (Fig. 5C), further supporting the active conformation of LRRK2^cent^ KIN domain.

Comparing the active conformations of LRRK2^cent^ and LRRK2^RCKW^–DNL201, we observed several common features. The αC helix, activation loop, APE-αF, and αH-αI linkers from the KIN domain are the major contributors for interactions with COR, and the interface between the KIN and COR domains are almost identical (Fig. 5D) despite a small displacement of COR domains (Fig. 5C). The seesaw motion between the COR-B and ROC domains is also observed, as seen by the Tyr^1699^ side-chain flipping (fig. S8A). However, these activation features are different from or were not observed in the previous microtubule-based LRRK2 model (PDB 6XR4) (fig. S8B) (11). Additionally, there are global differences between Rab29-dependent and microtubule-based activation of LRRK2 because the Rab29–LRRK2 tetramer contains asymmetric dimers and microtubule-based LRRK2 oligomers are symmetric (fig. S8, C and D).

The conformational changes revealed by comparing active LRRK2^cent^ or active-like LRRK2–DNL201 with inactive LRRK2 align very well with previous HDX-MS data (13–16). Overall, the active conformation has a more compact arrangement; the αC helix, activation loop, APE-αF loop of the KIN domain (Fig. 4F and Fig. 5D), and C-terminal part of COR-B helix (residues 1788 to 1797) showed lower deuterium exchange (14, 15), owing to the closure of the central cavity upon activation (Fig. 3E). The only exception was the C-terminal half of the αC helix of ROC domain (residues 1426 to 1449), which became more accessible and showed increased deuterium exchange because of the seesaw motion (fig. S5K).

## Rab29–LRRK2 tetramer and Rab29-dependent activation

As the Rab29–LRRK2 tetramer has two protomers in active kinase conformation, we hypothesized that the tetrameric assembly could explain the increased Rab29-induced LRRK2 Ser^1292^ autophosphorylation (28). To test the hypothesis, we first verified that the tetramer state was not caused by the Rab29 Q67L mutation, which disrupts Rab29 membrane localization and impacts the Rab29-dependent LRRK2 activation in cells (23, 27). Thus, we characterized the Rab29 T71A/S72A–LRRK2 complex because Rab29 T71A/S72A has minimal impacts on LRRK2 cellular localization or kinase activity (fig. S9, A to D) (28). Our cryo-EM analysis showed that Rab29 T71A/S72A–LRRK2 forms tetramers during 2D classification performed with cross-correlation (fig. S9E), although there was a lower ratio of tetramer particles than with Rab29_EM_–LRRK2, likely because of a lower percentage of GTP-bound Rab29.

**Fig. 6.**
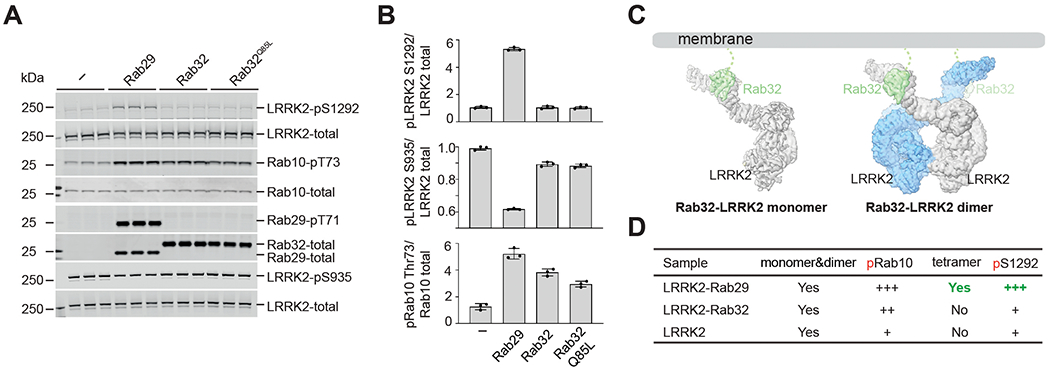
Rab29-LRRK2 tetramer and kinase activation. (**A** and **B**) Quantitative immunoblotting analysis of the cellular kinase activity of LRRK2 in the presence of Rab29 or Rab32. HEK293 cells were transiently cotransfected with WT LRRK2 and hemagglutinin (HA)–tagged empty vector (“–”), HA-tagged Rab29, or HA-tagged Rab32 (WT or Q85L mutant). Data are presented as ratios of pLRRK2-Ser^1292^/total LRRK2, pRab10-Thr^73^/total Rab10, and pLRRK2-Ser^935^/total LRRK2, normalized to the average of LRRK2 WT values. The data shown are the mean ± SD of three determinations. (**C**) Cryo-EM maps of the Rab32–LRRK2 complex. (**D**) Summary of LRRK2 kinase activity and LRRK2 states observed in the cryo-EM study of LRRK2 alone, in the presence of Rab32, or in the presence of Rab29.

We then compared Rab29–LRRK2 and Rab32–LRRK2 complexes to dissect the role of LRRK2 tetramerization in kinase activation. Rab32, a close homolog of Rab29 (~56% sequence identity and ~70% similarity) can mediate LRRK2 membrane recruitment (31) but does not support LRRK2 activation in human embryonic kidney 293 (HEK293) cells, as indicated by low Ser^1292^ autophosphorylation levels (28) and by a higher level of Ser^935^ phosphorylation (Fig. 6, A and B), which is associated with inactive LRRK2 (41). We determined the cryo-EM structure of Rab32–LRRK2 complexes (fig. S10, A to C). Rab32 interacts with LRRK2 through an interface that is almost identical to that used by Rab29 (fig. S10, D and E), but the Rab32–LRRK2 complex was captured in two oligomerization states: Rab32–LRRK2 monomer and Rab32–LRRK2 dimer (Fig. 6C). Furthermore, reprocessing of our previous LRRK2-alone dataset (10) showed no LRRK2 tetrameric assembly (Fig. 6D and fig. S10F). Therefore, we conclude that the Rab29–LRRK2 tetramer is associated with the Rab29-dependent activation of Ser^1292^ autophosphorylation.

In contrast to our results for Ser^1292^ autophosphorylation, we found that phosphorylation of Rab10 by LRRK2 is stimulated by both Rab29 and Rab32 (Fig. 6B), suggesting that membrane recruitment of LRRK2 is sufficient to activate Rab10 phosphorylation without a requirement for tetramerization. Rab10 phosphorylation and Ser^1292^ autophosphorylation are thus independent molecular events during LRRK2 signaling (42, 43). Endogenous Rab38 was also reported to increase Rab10 phosphorylation but not Ser^1292^ autophosphorylation in melanocytes (44). Therefore, we predict that Rab38 should mediate LRRK2 membrane recruitment but not the tetramerization of LRRK2.

## Discussion

In this study, we have presented Rab29–LRRK2 structures in both active and inactive states, which allowed us to analyze PD mutations in the context of kinase activation. Gain-of-function mutations at six sites—G2019S, I2020T, Y1699C, N1437H, R1441C/G/H, and S1761R (fig. S11A)—have been proposed to be high risk and PD-causing (45). Our previous structure of LRRK2 bearing the G2019S substitution showed little difference from the wild-type (WT) LRRK2 in the inactive state (10). However, this mutation could induce additional interactions with Glu^1920^ and stabilize the critical Lys^1906^-Glu^1920^ salt bridge in the active conformation (fig. S11C). Ile^2020^ moves from a hydrophobic to a hydrophilic environment upon LRRK2 activation (fig. S11B), and the I2020T mutation would destabilize the inactive conformation and favor the active conformation. Tyr^1699^, Asn^1437^, and Arg^1441^ are at the interface between the αC helix of ROC and the COR-B domain, where a seesaw-like motion of the ROC-αC helix occurs upon LRRK2 activation (fig. S11D, fig. S5K, and movie S2). Asn^1437^ and Arg^1441^ are located at one side of the seesaw and anchor the C-terminal part on the αC helix of ROC to the surface of the COR-B domain in the inactive state (fig. S11D). Therefore, mutations of Asn^1437^ and Arg^1441^ should weaken the anchoring effect, shifting the balance of the seesaw toward the N-terminal of the ROC αC helix, hence promoting LRRK2 activation. Tyr^1699^ functions as the pivot point (fig. S11D and movie S2), and its substitution with a smaller residue would lower the energy barrier for the seesaw motion and for the transition from the inactive to the active state. The above structural observations lead us to conclude that increased conformational dynamics of G2019S, I2020T, Y1699C, N1437H, and R1441C/G/H mutations play an important role in PD pathogenesis.

The different modes of activation of LRRK2 with Rab29 or its close homologs, Rab32 or Rab38, are intriguing. Although Rab29, Rab32, and Rab38 could all activate Rab10 phosphorylation by LRRK2, only Rab29 promotes the formation of LRRK2 tetrameric assembly, boosts Ser^1292^ autophosphorylation, and is associated with late-onset PD. Moreover, LRRK2 Ser^1292^ autophosphorylation is elevated in urinary exosomes of *LRRK2* mutation carriers (46–48), but the association between Rab10 phosphorylation and *LRRK2* mutations varies across different studies (46–50). We thus speculate a pathogenic association between Rab29-dependent LRRK2 tetramerization and Ser^1292^ autophosphorylation in patients with *LRRK2* mutations. It would also be interesting to examine the Ser^1292^ autophosphorylation in PD patients with *PARK16* variations.

LRRK2 activation features a striking dimer-of-asymmetric dimer assembly containing two active core subunits encased by two inactive peripheral protomers. Intermolecular interactions between two asymmetric LRRK2 dimers stabilize the active Rab29–LRRK2 tetramer (Fig. 3, A to D). The formation of active tetramers is Rab29-dependent because such an assembly was not observed with Rab32 or without Rab29 under similar experimental conditions (Fig. 6, C and D) (10). Furthermore, Rab29 binding to the extended ARM-ANK-LRR portion of LRRK2^cent^ appears to unlock or facilitate LRRK2^cent^ activation (fig. S5A). However, the low resolution of LRRK2^cent^ ARM-ANK-LRR domains prevents us from dissecting these putative mechanisms in atomic details. Nevertheless, this Rab29- and oligomerization-controlled asymmetric activation of LRRK2 adds a new mode of kinase asymmetric activation, currently represented by EGFR (51), IRAK4 (52), and B-Raf (53, 54).

Lastly, LRRK2 activation is clearly a complex process and could be achieved by other mechanisms, such as lipid oxidation, Rab12, and microtubule-based filamentation (11, 12, 55–58). This study focuses on Rab29-induced LRRK2 activation and provides a framework for interpreting disease mutations in the context of kinase activation. Our data suggest that allosteric inhibition of LRRK2 could be potentially achieved by disrupting Rab29–LRRK2 interaction, blocking LRRK2 oligomerization, or preventing the conformational transition from the inactive to the active states. Thus, our findings provide novel insights into LRRK2-based drug development and PD treatment.

## Supplementary Material

Movie S1

Movie S2

Supplementary Material

## Data Availability

The cryo-EM maps of Rab29-LRRK2 monomer, dimer, tetramers and LRRK2^RCKW^-DNL201 complexes have been deposited in the Electron Microscopy Data Bank under accession codes EMD-29339, EMD-29341, EMD-29342, and EMD-40588. The corresponding coordinates have been deposited in the Protein Data Bank under accession codes 8FO2, 8FO8, 8FO9, and 8SMC, respectively. Raw micrographs of the Rab29-LRRK2 complex have been deposited in the Electron Microscopy Public Image Archive (EMPIAR) with accession code 47485217. Plasmids encoding constructs used for cryo-EM in this study are available upon request. All the primary immunoblotting and confocal microscopy data that are presented in this study have been deposited at Zenodo (59). Plasmids and antibodies (and associated datasheets) generated at the MRC PPU at the University of Dundee can be requested through our website: https://mrcppureagents.dundee.ac.uk/.
