# Supplementary material for: Rab29-dependent asymmetrical activation of leucine-rich repeat kinase 2
Source: Science. Author manuscript; Available in PMC 2024 Jan 12. (PMC10786121; doi:10.1126/science.adi9926)
Supplement: Supplementary Material [file NIHMS1954122-supplement-Supplementary_Material.pdf]

Supplementary Information

Rab29-dependent asymmetrical activation of leucine-rich repeat kinase 2

Hanwen Zhu[1*], Francesca Tonelli[3,4*], Martin Turk[2], Alan Prescott[3], Dario R. Alessi[3,4], Ji Sun[1,†]

[1]Department of Structural Biology, St. Jude Children's Research Hospital, Memphis, TN 38105, USA

[2]Cryo-EM and Tomography Center, St. Jude Children's Research Hospital, Memphis, TN 38105, USA

[3]MRC Protein Phosphorylation and Ubiquitylation Unit, School of Life Sciences, University of Dundee, Dundee DD1 5EH, United Kingdom

[4]Aligning Science Across Parkinson's (ASAP) Collaborative Research Network, Chevy Chase, MD, USA.

[*]These authors contributed equally to this work.

[†]Correspondence: ji.sun@stjude.org (J.S.)

Materials and Methods

Cloning, expression and purification of human LRRK2 and Rab29

The cDNA encoding full-length human LRRK2 was purchased from Horizon Discovery and contained three natural variants (R50H, S1647T and M2397T) from the canonical LRRK2 sequence (NP_940980.4). The $LRRK2^{RCKW}$ was subcloned from the pDEST53-LRRK2-WT vector (Addgene: 25044), and the M1732R mutation was introduced for structural and biochemical studies using the QuickChange Site-Directed Mutagenesis kit (Strategene) to prevent protein aggregation (*10*). A GFP tag followed by a preScission protease cleavage site was engineered at the N terminus of full-length LRRK2 or $LRRK2^{RCKW}$, which was cloned into the BacMam expression vector (*60*). Recombinant baculoviruses of full-length LRRK2 or $LRRK2^{RCKW}$ were generated using the Bac-to-Bac system according to the manufacturer's instructions (Invitrogen). Then P3 virus of full-length LRRK2 or $LRRK2^{RCKW}$ was used to transfect HEK293F cells for protein expression. Briefly, for 1 L cultures of HEK293F cells (~2-3x10^6 cells/mL) in Freestyle 293 media (Gibco) supplemented with 2% FBS (Gibco), about 100 mL P3 virus was used. Infected cells were incubated at 37°C overnight, and protein expression was induced by adding 10 mM sodium butyrate. Cells were cultured at 30°C for another 48-60 h before harvest. For full-length LRRK2 purification, the cell pellet from the 600 mL culture was resuspended in 30 mL lysis buffer (20 mM Tris pH 8.0, 200 mM NaCl, 5% glycerol, 2 mM DTT and protease inhibitors), and then cells were lysed by brief sonication. LRRK2 was separated from the insoluble fraction by high-speed centrifugation (38,000 × g for 1 h), and incubated with 1 mL CNBr-activated sepharose beads (GE Healthcare) coupled with 1 mg high-affinity GFP nanobodies (GFP-NB) (*61*). The GFP tag was cleaved by preScission protease at 4°C, and LRRK2 was further purified by size-exclusion chromatography with a Superose 6 Increase 10/300 GL column (GE Healthcare) equilibrated with 20 mM Tris pH 8.0, 200 mM NaCl and 2 mM DTT. The purified protein was collected and concentrated to 13 mg/ml (OD_{280}) using a 100-kDa MWCO centrifugal device (Ambion), flash-frozen in liquid N_2 and stored at -80°C. As for $LRRK2^{RCKW}$, the cell pellet from the 600 mL culture was resuspended in 30 mL lysis buffer (20 mM HEPES pH 7.4, 300 mM NaCl, 5% glycerol, 2 mM DTT and protease inhibitors), and then cells were lysed by brief sonication. $LRRK2^{RCKW}$ was separated from the insoluble fraction by high-speed centrifugation (38,000 × g for 1 h) and incubated with 1 mL CNBr-activated sepharose beads (GE Healthcare) coupled with 1 mg high-affinity GFP nanobodies (GFP-NB) (61). The GFP tag was cleaved by preScission protease at 4°C, and $LRRK2^{RCKW}$ was further purified by size-exclusion chromatography with a Superose 6 Increase 10/300 GL column (GE Healthcare) equilibrated with 20 mM HEPES pH 7.4, 200 mM NaCl and 2 mM DTT. The purified protein was collected and concentrated to 2.3 mg/ml (OD_{280}) using a 100-kDa MWCO centrifugal device (Ambion), flash-frozen in liquid N_2 and stored at -80°C.

The cDNA encoding human Rab29 and Rab32 were purchased from Horizon Discovery and synthesized by Integrated DNA Technologies, respectively. Residues 1-177 of Rab29 or 1-198 of Rab32 were cloned into the modified pGEX plasmid (Sigma), which attaches a GST tag and a TEV protease cleavage site at the N-terminus. For structural and biochemical studies, we generated $Rab29_{EM}$ or Rab32 Q85L mutant, which was constructed using the QuickChange Site-Directed Mutagenesis kit (Strategene). The recombinant proteins were overexpressed in *Escherichia coli* strain BL21 (DE3) in LB media supplemented with 0.05 mg/ml ampicillin, and cells were grown at 37°C until OD_{600} reached 0.8. Further expression was induced by adding 0.4 mM IPTG, and cells were allowed to grow at 16°C for 20 h. Harvested cells were lysed by sonication in the lysis buffer (20 mM Tris-HCl pH 8.0, 200 mM NaCl, 5% glycerol, 5 mM $MgCl_2$, and 1 mM PMSF), and lysates were cleared by centrifugation at 38,000 × g for 45 min. Subsequently, the proteins were purified by GST affinity chromatography on the glutathione Sepharose beads (GE Healthcare) in lysis buffer. The bound proteins were then eluted with lysis

buffer containing 20 mM GSH and incubated with 1 mM GppNHp at 4°C overnight, followed by gel filtration chromatography using a Superdex 200 Increase 10/300 GL column (GE Healthcare) in a storage buffer (20 mM Tris-HCl pH 8.0, 100 mM NaCl, 1 mM $MgCl_2$, and 2 mM DTT). For the structural study, the GST tag was removed by on-column cleavage with TEV protease at 4°C overnight before gel filtration chromatography. The peak fractions were collected and concentrated to 16 mg/ml (OD_{280}), flash-frozen in liquid N_2, and stored at -80°C.

Cryo-EM sample preparation

Cryo-EM grids were prepared with a Vitrobot Mark IV (FEI). Quantifoil R1.2/1.3 300 Au holey carbon grids (Quantifoil) were glow-discharged for 30 s. Purified full-length LRRK2 and Rab29 or Rab32 were incubated together on the ice for 1 h with a final concentration of 30 μM and 200 μM in the presence of 2 mM ATP, 1 mM GppNHp and 1 mM $MgCl_2$. In addition, 2.3 mM fluorinated Fos-Choline-8 was added right before freezing the grids, and then 3.0 μL of protein sample were pipetted onto the grids, which were blotted for 5 s under blot force -3 at 16°C and 95% relative humidity and plunge-frozen in liquid nitrogen-cooled liquid ethane. For cryo-EM sample preparation of $LRRK2^{RCKW}$ in the presence of DNL201 (Medkoo Biosciences, Inc.), the purified $LRRK2^{RCKW}$ (2.3 mg/ml) was incubated with 100 μM DNL201 on ice for 30 min and prior to vitrification centrifuged at 4°C (38,000 × g for 10 min) to remove potential precipitation. 2.3 mM fluorinated Fos-Choline-8 was added right before freezing the grids, followed by a double application of 3.0 μL of protein sample onto the grids with a manual first blotting step, with a 3.5-s blot time, blot force of -3 under 16°C and 95% relative humidity after second sample loading and plunge-frozen in liquid nitrogen-cooled liquid ethane.

Cryo-EM data acquisition and processing

The Rab29-LRRK2 dataset was collected on a Titan Krios (Thermo Fisher Scientific) transmission electron microscope equipped with a K3 direct electron detector and post-column GIF energy filter (Gatan). Data collection was performed in an automated manner using EPU (Thermo Fisher Scientific). Movies were recorded at defocus values from -0.6 to -1.8 μm at a magnification of 81kx in super-resolution mode, corresponding to a pixel size of 0.53 Å. During 3.0 s exposure, 60 frames were collected with a total electron dose of ~59 $e^-/Å^{-2}$ (at a dose-rate of 0.9795 $e^-/frame/Å^2$). In total, 11,762 images were collected. Motion correction was performed on raw super-resolution movie stacks and binned by 2 using MotionCor2 (62). Contrast transfer function (CTF) estimation was performed using Gctf (*63*). Prior to particle picking, micrographs were analyzed for good power spectrum, and the bad ones were discarded (with 11,260 good images remaining).

Particles were selected using the template picker in cryoSPARC (*64*) and extracted using a binning factor of 2. Several rounds of the 2D classification were performed to eliminate ice artifacts, carbon edges, and false-positive particles containing noise. During 2D classification, three groups of classes were observed, corresponding to the LRRK2 monomer, dimer and tetramer states, respectively. All groups were selected, and *ab initio* reconstruction was performed. In order to further separate the LRRK2 monomer, dimer, and tetramer states, we performed Heterogeneous refinement in cryoSPARC. As a result, 104,718 particles were assigned to the monomer class, 86,379 particles to the dimer, and 62,946 particles to the tetramer. All 3D classes were further refined using cryoSPARC after extraction of unbinned particles corresponding to each identified sub-set. For the LRRK2 monomer state, we performed a standard NU-refinement without

imposing symmetry, followed by a focused 3D refinement (with a soft mask around Rab29-LRRK2 interface region) to improve the map quality of the ARM domain of LRRK2 with Rab29 binding. For the LRRK2 dimer state, we performed NU-refinement by applying C2 symmetry. For the LRRK2 tetramer state, in addition to NU-refinement with imposed C2 symmetry, we also performed symmetry expansion to further improve the resolution of $LRRK2^{peri}$ and focused refinement to better resolve regions of $LRRK2^{cent}$. Local and global CTF refinements were performed to correct lens aberrations and improve the resolution of the final reconstruction. All resolution estimates were calculated according to the gold-standard Fourier shell correlation (FSC) using the 0.143 criterion (*65*). Local resolution was estimated in cryoSPARC. The density maps were B-factor sharpened in cryoSPARC and used to produce figures.

The Rab32-LRRK2 dataset was collected on a Talos Arctica (Thermo Fisher Scientific) operated at 200 kV, equipped with a K3 Summit direct electron detector and a post-column GIF energy filter (Gatan). Automated data collection was performed using EPU (Thermo Fisher Scientific). Movies were recorded at defocus values from -1.0 to -2.4 μm at a magnification of 79kx, which corresponded to a pixel size of 1.044 Å at the specimen level (super-resolution 0.522 Å/px). During 4.0 s exposure, 60 frames were collected, corresponding to a total electron dose of ~60 e^-/$Å^{-2}$ (at a dose rate of 0.9943 e^-/frame/$Å^2$). In total, 5,966 images were recorded. Motion correction, CTF estimation, and image selection were performed as described for the Rab29-LRRK2 dataset. After selection, 5,476 images remained.

The Rab32–LRRK2 dataset was processed similarly, except for the following differences. Particles were selected using the template picker in cryoSPARC (*64*) and extracted using a binning factor of 3. Several rounds of 2D classification were performed to eliminate ice artifacts, carbon edges, and false-positive particles containing noise. During 2D classification, two groups of classes were observed corresponding to the LRRK2 monomer and dimer states. Both groups were selected, and *ab initio* reconstruction was performed. In order to further separate the LRRK2 monomer and dimer states, we performed Heterogeneous refinement in cryoSPARC. As a result, 41,831 particles were assigned to the monomer class and 39,337 particles to the dimer. All 3D classes were refined in parallel using cryoSPARC after extraction of corresponding particles by binning 2. For the LRRK2 monomer state, we performed a standard NU-refinement by applying C1 symmetry followed by a focused 3D refinement step to improve the map quality of the ARM domain of LRRK2 with Rab32 binding. For the LRRK2 dimer state, we performed NU-refinement by applying C2 symmetry. All resolution estimates were calculated according to the gold-standard Fourier shell correlation (FSC) using the 0.143 criterion (*65*). The density maps sharpened in cryoSPARC were used to produce figures.

The $LRRK2^{RCKW}$–DNL201 dataset was collected as described for the Rab29-LRRK2 dataset. Movies were recorded at defocus values from -1.0 to -2.8 μm at a magnification of 130kx in hardware binning mode, corresponding to a pixel size of 0.6485 Å at the specimen. During 1.8 s exposure, 60 frames were collected with a total electron dose of ~69 e^-/$Å^{-2}$ (at a dose-rate of 1.2 e^-/frame/$Å^2$). In total, 33,456 images were collected. Motion correction, CTF estimation and image selection were performed as described for the Rab29-LRRK2 dataset. After the selection of high-quality micrographs, 33,408 images were used during the data process.

Particles were first selected using the Blob picker in cryoSPARC (*64*) and extracted using an initial binning factor of 6. Several rounds of 2D classification were performed to eliminate ice artifacts, carbon edges, and false-positive particles containing noise. The initial 2,432 good particles from 2,279 micrographs were selected to create a training dataset for the Topaz neural network-based picker (*66*). These particles were then used to train a Topaz model that was used for particle picking from all micrographs. Topaz was run in training mode using a downsampling factor of 4

and an estimated number of particles per micrograph of 15. The resulting model was used to pick particles using Topaz Extract, followed by extraction from micrographs. Duplicate particles were removed, and multiple rounds of 2D classification were performed to isolate homogeneous subsets. To further sort the dataset into more homogeneous subsets, we performed rounds of Heterogeneous Refinement and NU refinement without imposing symmetry in cryoSPARC. The best class contained 59,515 particles and yielded a 4.0 Å overall resolution map (fig. S6C). All resolution estimates were calculated according to the gold-standard Fourier shell correlation (FSC) using the 0.143 criteria (65). Local resolution was estimated in cryoSPARC.

Cross-correlation analysis of 2D class averages and reconstructed cryoEM maps

Particles from the Rab29 T71A/S72A-LRRK2 cryo-EM dataset were subjected to several rounds of reference-free classification, eliminating edge artifacts and ice contaminants from the particle-picking process. Based on the size, 2D class averages were grouped into three subsets, followed by another round of 2D classification. These sub-sets of reference-free 2D class averages were then compared with projections of the cryoEM maps from the $Rab29_{EM}$-LRRK2 cryo-EM dataset. From each cryoEM map (monomer, dimer and tetramer), stacks of 200 projection images were generated in cryoSPARC. The similarity between 2D class averages and map projections was measured by cross-correlation. For every 2D class average, a cross-correlation score was calculated with every projection at an angular step size of 5 degrees. The highest scoring projection within each stack corresponds to the best matching map orientation to a particular 2D class average (for tetramer, fig. S9E). The cross-correlation analysis was implemented in Python using the signal correlate module part of the SciPy library, NumPy, and a custom module for reading mrc files.

Model building and refinement

The reported structures of LRRK2 (PDB 7LI4 and 7LHT) (*10*) and the predicted structures of LRRK2 and Rab29 by AlphaFold2 (67) were fitted and adjusted into the cryo-EM maps of the Rab29-LRRK2 monomer, dimer, and tetramer states using Chimera (*68)* and Coot (*69*). The reported structures of LRRK2 (PDB 7LI4 and 7LHT) (*10*) and Rab32 (PDB 6FF8) (*25*) were fitted and adjusted into the cryo-EM maps of Rab32-LRRK2 in both monomeric and dimeric states using Chimera (*68*) and Coot (*69*). For the structural model of LRRK2 bound with DNL201, the central LRRK2 molecule of the tetrameric Rab29-LRRK2 was fitted and adjusted into the cryo-EM map using Chimera (*68*) and Coot (*69*). The structural model was refined against the map using the real space refinement module with secondary structure and non-crystallographic symmetry restraints in the Phenix package (*70*). Fourier shell correlation curves were calculated between the refined model and the full map. The geometries of the models were validated using MolProbity (*71*). All the figures were prepared in PyMOL (Schrödinger, LLC.), UCSF Chimera (*68*) and UCSF ChimeraX (*72*).

GST pull-down assay

Twenty micrograms (or 0.4 nmol) of WT or various mutants of GST-tagged Rab29 proteins were immobilized onto 30 μL of the glutathione Sepharose beads (GE Healthcare) and then incubated

with 870 μg (or 3 nmol) LRRK2 proteins in 1 mL binding buffer containing 20 mM Tris-HCl pH 8.0, 100 mM NaCl, 10% glycerol, 1 mM GTP, 5 mM $MgCl_2$, 2 mM DTT and protease inhibitors for 3 h at 4°C. The beads were washed three times (10 min each) with binding buffer, and bound proteins were eluted with binding buffer supplemented with 20 mM GSH. The protein samples were subjected to SDS-PAGE followed by Coomassie brilliant blue staining. GST was used as the control.

Measurement of DNL201's inhibition of LRRK2 in vitro

The LRRK2 inhibition assay was performed using a commercially available ADP-Glo™ Kinase Assay kit (Promega). Dilutions of DNL201 were prepared by serial dilution of a 50 mM stock solution of DNL201 in DMSO into ddH_2O. The standard LRRK2 kinase reaction solution (5 μl) was consisting of 40 mM Tris-HCl pH7.4, 20 mM $MgCl_2$, 0.1 mg/ml BSA, 2 mM DTT, 10 μM ATP, 190 μM LRRKtide, and varied concentrations of the DNL201 inhibitor, and the reaction was initiated by the addition of 5 nM $LRRK2^{RCKW}$ enzyme followed by incubation at room temperature for 100 min. Then 5 μl ADP-Glo™ reagent was added to the mixture to stop the kinase reaction and deplete unreacted ATP by further incubation at room temperature for 45 min. Afterwards, 10 μl of Kinase Detection reagent was added to convert ADP to ATP and introduce luciferase and luciferin to detect ATP by incubating at room temperature for 30 min. The luminescence signal was recorded at 520 nm with an integration time of 1-second in a Nunc 384-well plate using a POLARstar Omega microplate reader. The percentage of kinase activity compared to control without DNL201 inhibitor was plotted against the log concentration of the DNL201 inhibitor, and the data were fitted to a four-parameter logistic curve in GraphPad Prism version 8 (GraphPad, San Diego, CA). All experiments were performed for at least three times, and the data were presented as mean ± SD.

Plasmids used for cell-based assays

All plasmids used for cell-based assays were generated by the MRC PPU Reagents and Services at the University of Dundee (https://mrcppureagents.dundee.ac.uk). Each plasmid was confirmed by sequencing at the MRC Sequencing and Services (https://www.dnaseq.co.uk). All plasmids are available to request via the MRC PPU Reagents and Services website (https://mrcppureagents.dundee.ac.uk). HA-empty vector (DU49303); HA-Rab29 wild-type (DU50222); HA-Rab29 L7Q (DU72178); HA-Rab29 D43A (DU72179); HA-Rab29 W62A (DU 72177); HA-Rab29 L76M (DU 72180); HA-Rab29 Q67L (DU50241); HA-Rab29 T71A/S72A (DU52670); HA-Rab29 Q67L/T71A/S72A (DU72804); HA-Rab32 wild-type (DU52622); HA-Rab32 Q85L (DU77209); Flag-LRRK2 wild-type (DU62804); Flag-LRRK2 D2017A (kinase dead) (DU10128); Flag-LRRK2 R399E (DU72192); Flag-LRRK2 M402A (DU72193); Flag-LRRK2 L403E (DU72194); Flag-LRRK2 W1791A (DU72200); Flag-LRRK2 N1710A (DU72212); Flag-LRRK2 P1588A (DU72201); GFP-LRRK2 wild-type (DU72507); GFP-LRRK2 R399E (DU72508); GFP-LRRK2 M402A (DU72578); GFP-LRRK2 L403E (DU72579).

Cell transfection and lysis

Cell transfection method(dx.doi.org/10.17504/protocols.io.bw4bpgsn) and cell lysis method (dx.doi.org/10.17504/protocols.io.b5jhq4j) have previously been described. Briefly, HEK293 cells (ATCC Cat no. CRL-1573, RRID:CVCL_0045) were seeded into 6-well plates and transiently transfected at 60-70% confluence using Polyethylenimine (PEI) transfection reagent (Polysciences, Inc., #24765). For the microscopy experiments, cells were plated on 22mm x 22mm glass coverslips sterilized in 100% ethanol for 30 min. For each well, 1.6 μg of N-ter Flag-tagged or GFP-tagged LRRK2 (wild-type or mutant), 0.4 μg of N-ter HA-tagged Rab29 (wild-type or mutant) (or HA-empty vector) and 6 μg of PEI were diluted in 0.5 mL of Opti-MEM™ Reduced serum medium (Gibco™) and incubated for 15-20 min at room temperature before being added to the cells. For quantitative immunoblot analysis, cells were lysed 24 h post-transfection in ice-cold lysis buffer containing 50 mM Tris-HCl pH 7.4, 1 mM EGTA, 10 mM 2-glycerophosphate, 50 mM sodium fluoride, 5 mM sodium pyrophosphate, 270 mM sucrose, supplemented with 1 μg/ml microcystin-LR, 1 mM sodium orthovanadate, complete EDTA-free protease inhibitor cocktail (Roche), and 1% (v/v) Triton X-100. For the experiment shown in fig. S3E, cells were treated with 200 nM MLi-2 or 0.1% (v/v) DMSO (vehicle) for 2 hours before lysis. Lysates were clarified by centrifugation at 17,000 × g at 4°C for 10 min and supernatants were quantified by Bradford assay. For fluorescence microscopy analysis, cells were fixed 24 h post-transfection in 4% (w/v) paraformaldehyde (PFS) in PBS, as described below.

Quantitative immunoblot analysis

A detailed description of our quantitative immunoblotting protocol has previously been described (dx.doi.org/10.17504/potocols.io.bsgrnby6). Briefly, cell lysates were mixed with a quarter of a volume of 4 x SDS-PAGE loading buffer (Invitrogen™ NuPAGE™ LDS Sample Buffer, cat# NP0007) and heated at 70°C for 5 min. Samples were loaded onto NuPAGE 4–12% Bis–Tris Midi Gels (Thermo Fisher Scientific, Cat# WG1402BOX or Cat# WG1403BOX) and electrophoresed at 130 V for 2 h in NuPAGE MOPS SDS running buffer (Thermo Fisher Scientific, Cat# NP0001-02). Proteins were then electrophoretically transferred onto a nitrocellulose membrane (GE Healthcare, Amersham Protran Supported 0.45 μm NC) at 90 V for 100 min on ice in transfer buffer (48 mM Tris and 39 mM glycine supplemented with 20% (v/v) methanol). The membranes were blocked with 5% (w/v) skim milk powder dissolved in TBS-T (50 mM Tris base, 150 mM sodium chloride (NaCl), 0.1% (v/v) Tween 20) at room temperature for 1 h before overnight incubation at 4°C in primary antibodies. Membranes were washed three times for 15 min each with TBS-T before being incubated with secondary antibodies for 1 h at room temperature. Thereafter, membranes were washed with TBS-T three times with a 15-min incubation for each wash, and protein bands were acquired via near-infrared fluorescent detection using the LI-COR Odyssey CLx Western Blot imaging system.

Antibodies used for quantitative immunoblotting analysis

The antibodies against Rab10 pThr73 [MJF-R21] (ab230261; RRID:AB_2811274), LRRK2 pSer1292 [MJFR-19-7-8] (ab203181; RRID:AB_2921223) and Rab29 pThr71 [MJF-R24-17-1] (ab241062; RRID:AB_2884878) were purchased from Abcam. The rabbit monoclonal antibody against LRRK2 pSer935 (UDD2; RRID:AB_2921228) was purified by MRC PPU Reagents and Services at the University of Dundee (https://mrcppureagents.dundee.ac.uk). The mouse monoclonal antibody against total LRRK2 (C-terminus) was purchased from NeuroMab (clone N241A/34, #75-253; RRID:AB_10675136). The mouse monoclonal antibody against total Rab10

was purchased from Nanotools (#0680–100/Rab10-605B11; RRID:AB_2921226). The anti-HA tag antibody was purchased from Sigma (cat #11867423001; RRID:AB_390918). All primary antibodies were diluted in 5% (w/v) bovine serum albumin (BSA) in TBS-T and used at a final concentration of 1:1,000 (v/v).

Goat anti-mouse IRDye 680LT (#926-68020; RRID:AB_10706161), goat anti-rabbit IRDye 800CW (#926-32211; RRID:AB_621843) and goat anti-rat IRDye 680LT (#926-68029; RRID:AB_10715073) IgG (H + L) secondary antibodies were from LI-COR Biosciences and were diluted 1:20,000 (v/v) in 5% (w/v) milk in TBS-T.

Fluorescence microscopy analysis

A detailed method is reported in dx.doi.org/10.17504/protocols.io.bp2l69r8klqe/v1. Briefly, cells were fixed in 4% (w/v) paraformaldehyde in PBS for 10 min at room temperature, followed by permeabilization with 0.1% (v/v) NP-40 in PBS for 10 min. Cells were then blocked in 1% BSA (w/v) in PBS for 1 h at room temperature. Blocked cells were incubated with primary antibodies for 1 h at room temperature. Cells were washed 3 times (10 min each) with 0.2% BSA (w/v) in PBS and incubated with secondary antibodies and 1 μg/ml DAPI (4′,6-diamidino-2-phenylindole, Dilactate) for 1 h at room temperature in the dark. Cells were washed 4 times (10 min each) with 0.2% BSA (w/v) in PBS and mounted on glass microscope slides using Vectashield mounting medium. Images were obtained using a Zeiss LSM 880 laser scanning microscopes using the Plan Apochromat ×63 objective (NA 1.4) objective with a zoom of 1.0 and optical section thickness of 0.8 μm (image size 1912 × 1912 pixels, pixel size 0.071 μm). Ten randomly selected fields with GFP-positive cells (i.e. cells that are successfully transfected with GFP-LRRK2) were collected for each sample, blinded to LRRK2 or Rab29 variant. Cells that were identified in both the LRRK2 (green) channel and Rab29 (red) channel (i.e. cells transfected with both LRRK2 and Rab29) were analysed for colocalization of red and green channels and Mander's correlation coefficient calculated indicating the proportion of LRRK2 overlapping with Rab29 above a specific threshold (set at 450). Cells not expressing both red and green were removed from analysis. >40 cells were analysed for each experimental condition. The Mander's correlation coefficients were analysed for statistical significance in GraphPad Prism 9.5.0 (RRID:SCR_002798) using a Kruskal-Wallis One-way ANOVA test after being tested for normal distribution.

Antibodies used for fluorescence microscopy analysis

Anti-HA tag mouse monoclonal antibody [HA.C5] (Abcam, ab18181; RRID:AB_444303) and anti-ACBD3 rabbit polyclonal antibody (Sigma-Aldrich, HPA015594; RRID:AB_1844491) were diluted in 0.2% BSA (w/v) in PBS at 1:1000 and 1:200 respectively.

Goat anti-Mouse IgG (H+L) Highly Cross-Adsorbed Alexa Fluor™ 568 (Invitrogen A11031; RRID:AB_14469) and Goat anti-Rabbit IgG (H+L) Highly Cross-Adsorbed Alexa Fluor™ Plus 647 (Invitrogen A32733; RRID:AB_2633282) secondary antibodies were diluted at 1:500 in 0.2% BSA (w/v) in PBS.

Quantification and Statistical Analysis

Immunoblotting data for the cell-based assays shown in Fig. 6A, fig. S3C-E, and fig. S9A were acquired using a LI-COR CLx Western Blot imaging system and quantified using Image Studio Lite (Version 5.2.5, RRID:SCR_013715). For data reported in Fig. 6A, the experiment was performed once (three independent biological replicates). For data reported in Fig. S3C-D, the experiment was performed once (three independent biological replicates). For data reported in Fig. S3E, the experiment was performed once (two independent biological replicates). For data reported in Fig. S9A, the experiment was performed twice (three independent biological replicates for each experiment). Data were plotted with GraphPad Prism 9.5.0 (RRID:SCR_002798). The graphs report individuals with mean and standard deviation. Quantified immunofluorescence microscopy data (Mander's coefficients) were plotted with GraphPad Prism 9.5.0. The quantification and statistical analyses for model refinement and validation were generated using MolProbity.

Supplementary figures

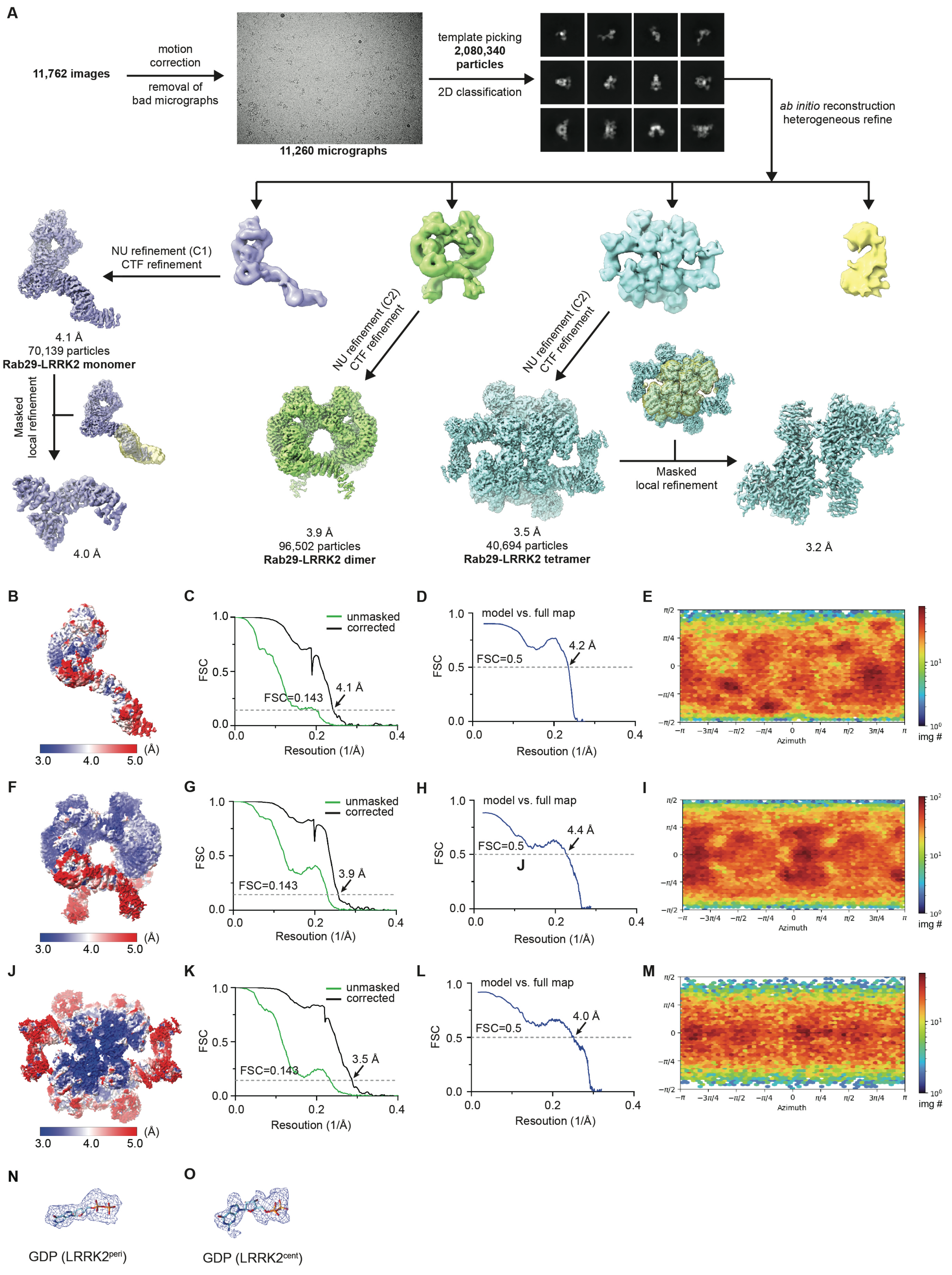

fig. S1: Structure determination of Rab29–LRRK2 complexes

(A) A simplified flow chart of cryo-EM data processing. **(B-O)** Structural validation of the cryo-EM analysis. Local resolution **(B,F and J)**, Fourier Shell Correlation (FSC) curves for the overall resolution **(C, G and K),** model-to-map fit between the full map and PDB coordinates **(D, H and L),** angular distribution calculated in cryoSPARC for particle projections (Heat map shows number of particles for each viewing angle) **(E, I and M)** of Rab29–LRRK2 complexes in the Rab29–LRRK2 monomer, dimer and tetramer states. **(N-O)** The density maps of the GDP bound to ROC domain of LRRK2^{peri} and LRRK2^{cent} in the Rab29-LRRK2 tetramer.

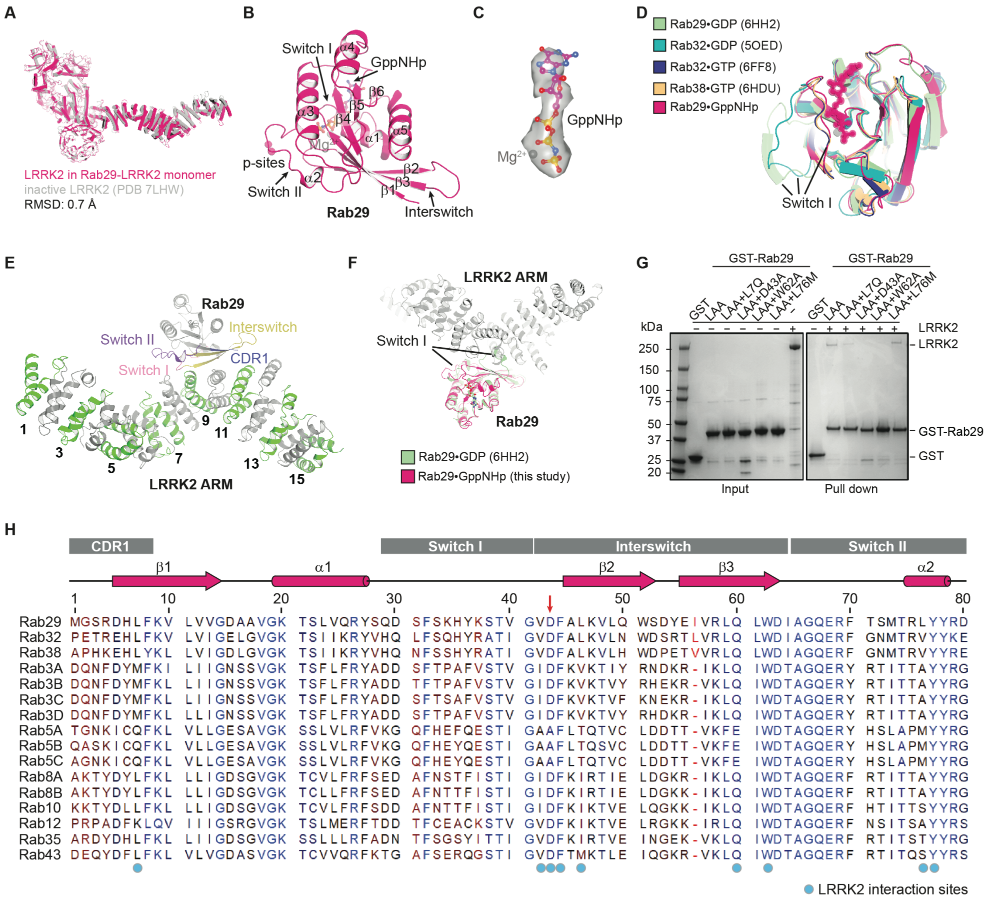

fig. S2: Structural features of the Rab29–LRRK2 monomer

(A) Structural comparison of inactive LRRK2-alone structure (PDB 7LHW) and Rab29-bound LRRK2 structure in the Rab29–LRRK2 monomer. **(B)** The cartoon model of Rab29 with secondary structures and key structural elements labeled. The phosphorylation sites (p sites) are shown as spheres. **(C)** Cryo-EM density of the nucleotide bound to Rab29 in the LRRK2 monomer state. **(D)** Superposition of LRRK2-bound Rab29 with other Rab proteins in GTP- and GDP-bound states. "Switch I" motifs are indicated. **(E)** Interactions between the LRRK2 ARM domains and Rab29. The ARM repeats of LRRK2 are indicated by numbers. **(F)** Superposition of Rab29 in the Rab29–LRRK2 complex with GDP-bound Rab29 (green). Potential steric clashes between GDP-bound Rab29 and LRRK2 are indicated. **(G)** *In-vitro* GST pull-down assays of Rab29 triple mutations (Q67L/T71A/S72A) or mutants (on the background of the triple mutation) and LRRK2. Gels were stained using Coomassie Blue. **(H)** Sequence alignment of Rab32 subfamily members

(Rab29, Rab32, and Rab38) and selective substrate Rabs of LRRK2. The residues involved in the interaction with LRRK2 are indicated with cyan circles. The secondary structures and "switch" motifs are also labeled. A red arrow indicates D43.

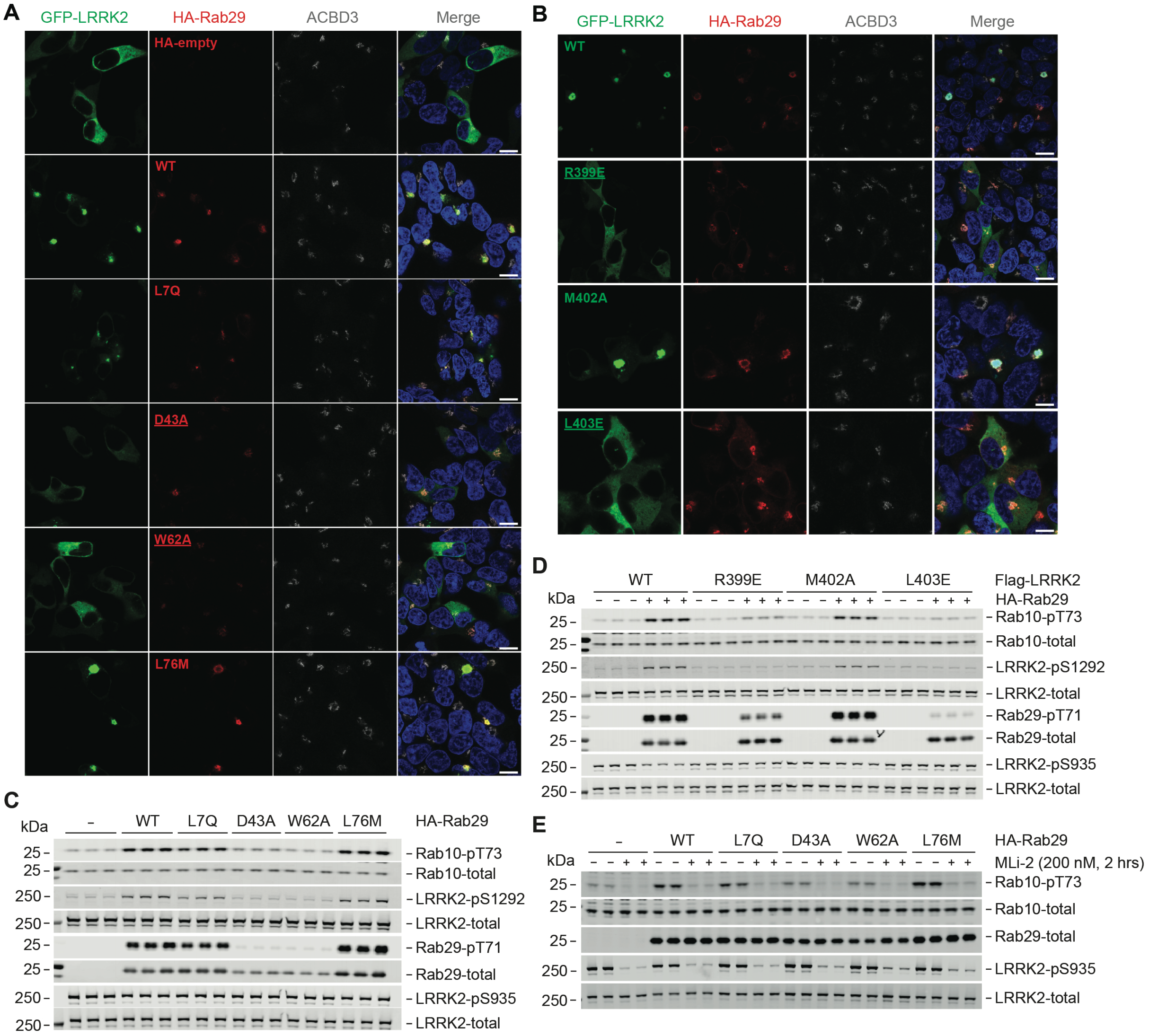

fig. S3: Functional analysis of Rab29-LRRK2 interface

(A) Wild-type GFP-LRRK2 (green) was co-expressed with wild-type or mutant (indicated) HA-Rab29 (red) in HEK293 cells followed by confocal microscopy localization analysis. **(B)** Wild-type or mutant (indicated) GFP-LRRK2 (green) was co-expressed with wild-type HA-Rab29 (red) in HEK293 cells followed by confocal microscopy localization analysis. The endogenous Golgi marker ACBD3 is also shown (white). Scale bar, 10 μm. Mutations that had significant impacts in Rab29 and LRRK2 colocalization are underlined. **(C-E)** Western blot of the kinase activity assay assessing impacts of Rab29-LRRK2 interface residues.

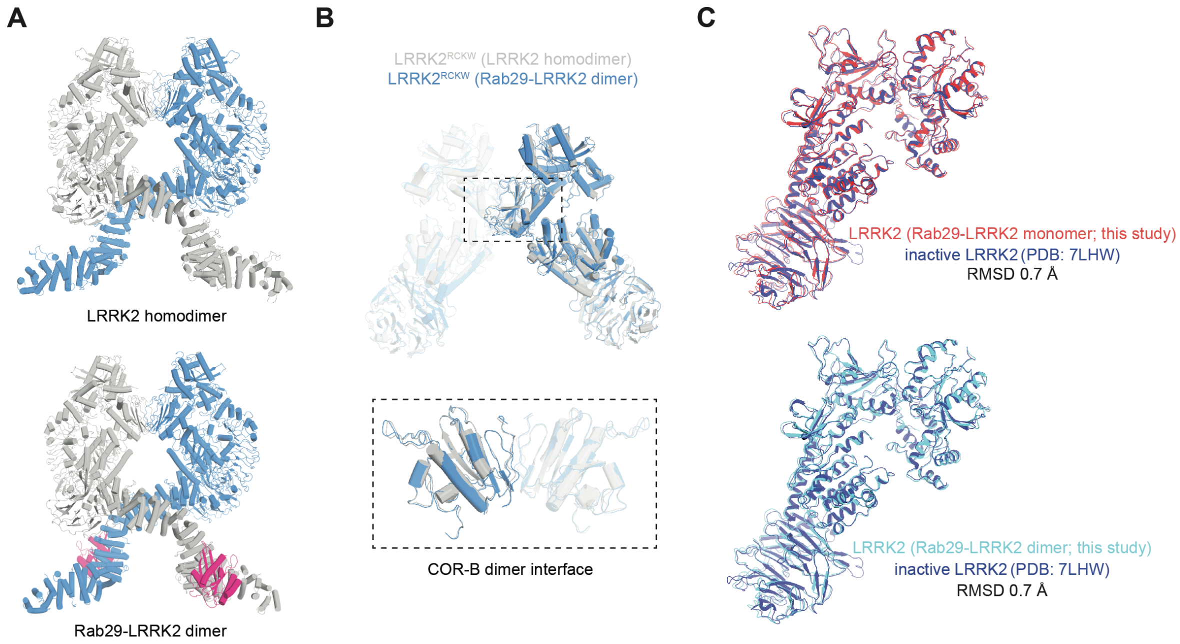

fig. S4: The Rab29–LRRK2 dimer

(A) Overall structures of LRRK2 homodimer and the Rab29–LRRK2 dimer. **(B)** LRRK2 comparison between homodimer and the Rab29–LRRK2 dimer, focusing on the RCKW part with COR-B dimer interfaces zoomed in. **(C)** The inactive conformation of LRRK2 in the Rab29–LRRK2 monomer and dimer states.

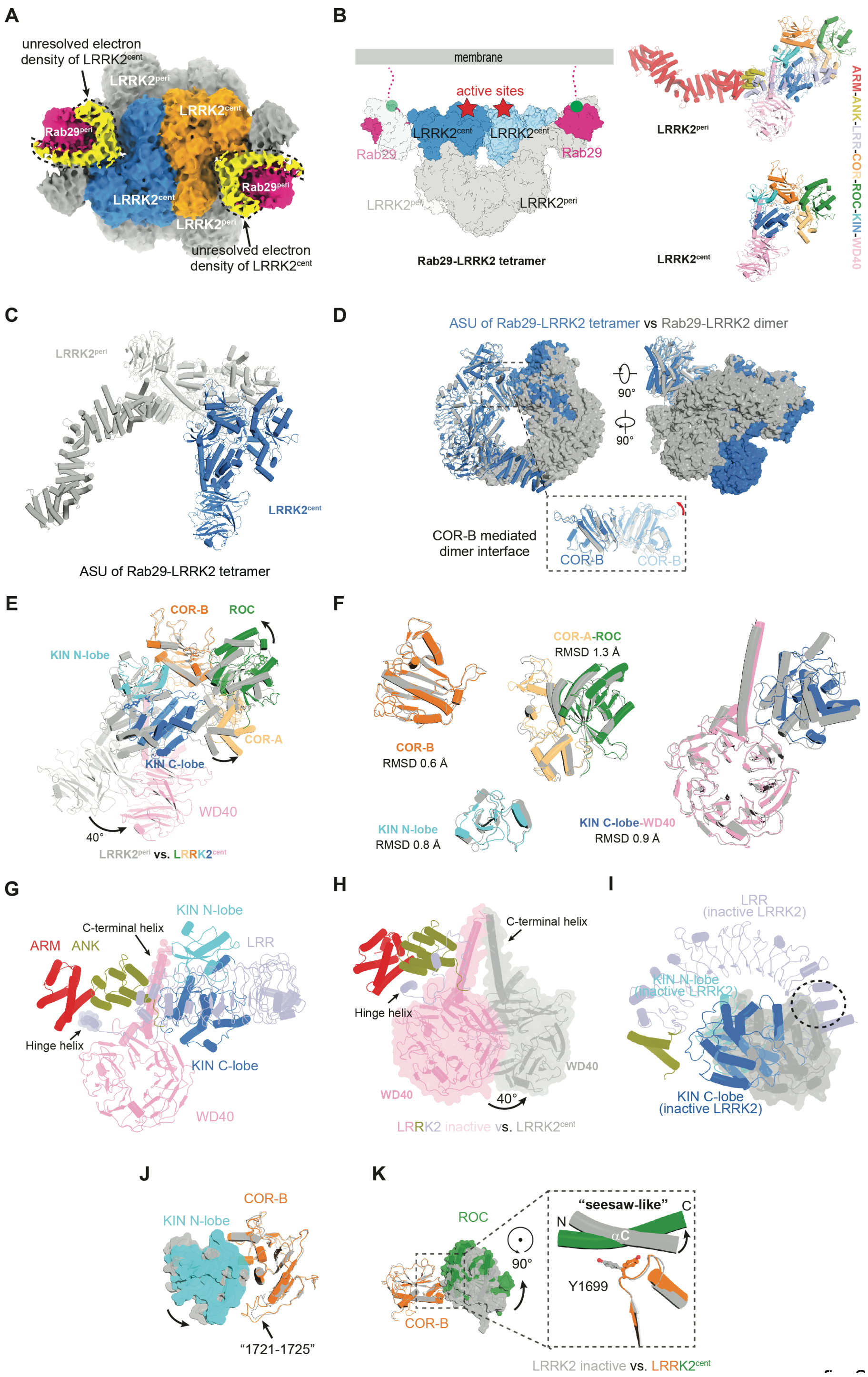

fig. S5: Structural features of the Rab29–LRRK2 tetramer

(A) Density map for the Rab29–LRRK2 complex in a low threshold, showing the flexible density of LRRK2^{cent} N-terminal domains (colored in yellow). **(B)** The two types of protomers in the Rab29–LRRK2 tetramer. Left: Rab29–LRRK2 tetramer shown as surface. Rab29, LRRK2^{cent}, and LRRK2^{peri} are colored in magenta, blue and grey, respectively. The membrane-anchoring N-terminal loops of Rab29 are indicated by dashed lines. Red stars mark active sites of LRRK2^{cent}.

Right: resolved models of LRRK2^{cent} and LRRK2^{peri}. **(C)** The asymmetric unit of the Rab29–LRRK2 tetramer. **(D)** Superposition of COR-B mediated LRRK2 dimer interfaces between Rab29–LRRK2 dimer and tetramer. **(E)** Superposition of the C-terminal catalytic halves of LRRK2^{cent} and LRRK2^{peri}. The conformational changes are indicated with arrowed lines. **(F)** Structural comparison of the rigid bodies in the C-terminal catalytic halves between inactive (grey) and active LRRK2 (colored). **(G)** Interdomain interactions in inactive LRRK2. The key scaffold elements: "hinge helix" and "C-terminal helix" are indicated. **(H-I)** The displacement of the WD40 domain and C-terminal helix upon LRRK2 activation and potential steric clashes upon activation (indicated by a dashed circle). **(J)** Comparison of the motion of COR-B and KIN N-lobe between the peripheral and central LRRK2 copies. The rotational movement of the KIN N-lobe towards COR-B and a loop (1721-1725) are indicated. **(K)** Comparison of ROC-COR-B domains between LRRK2^{cent} and LRRK2^{peri}. The rotational movement of the ROC domain relative to the COR-B subdomain is indicated. The proposed "seesaw-like" model constituted by Tyr1699 of the COR-B subdomain and αC helix of the ROC domain is shown in a zoom-in window.

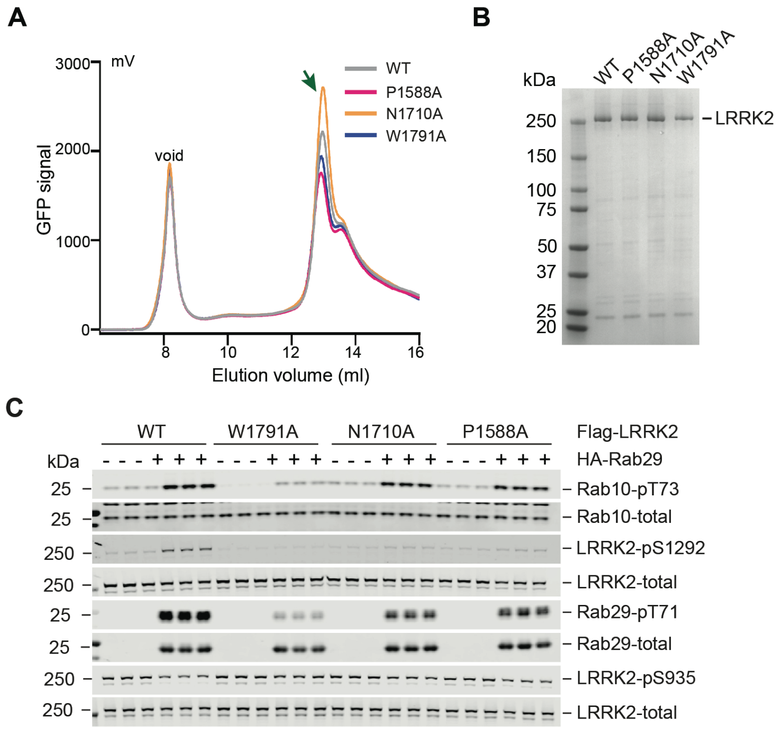

fig. S6: Biochemical characterization of KIN-COR interface residues.

(A) Fluorescence Detection Size Exclusion Chromatography (FSEC) assessment of KIN-ROC interface residues. All mutations migrate at the same position, like wild-type LRRK2, corresponding to monomers on the Superose-6 increase column. **(B)** SDS-PAGE of purified LRRK2 wild-type and mutation samples. **(C)** Western blot of the kinase activity assay assessing impacts of KIN-COR interface residues.

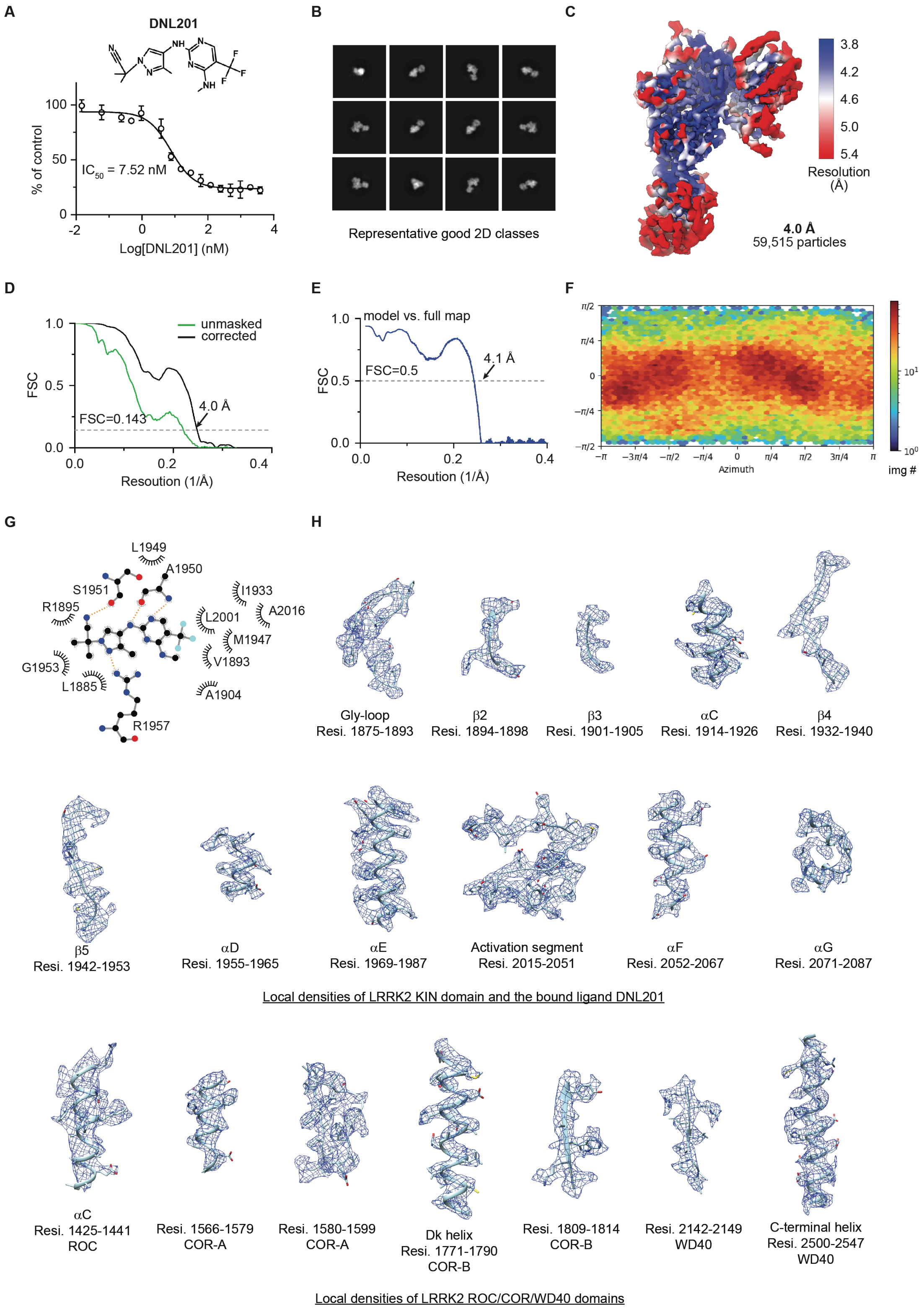

fig. S7: Cryo-EM analysis of LRRK2 with DNL201.

(A) Inhibition of the purified LRRK2^{RCKW} protein by DNL201. The data shown are the mean ± SD of three experiments. **(B)** Representative 2D classes of the LRRK2^{RCKW}–DNL201 complex from cryoSPARC. **(C)** Local resolution of the LRRK2^{RCKW}–DNL201 complex. **(D)** Fourier Shell Correlation (FSC) curves for the overall resolution of the LRRK2^{RCKW}–DNL201 complex. The golden standard (FSC=0.143) is used for resolution estimation. **(E)** Model-to-map fit between the full map and PDB coordinates of the LRRK2^{RCKW}–DNL201 complex. **(F)** Angular distribution calculated in cryoSPARC for particle projections. Heat map shows number of particles for each viewing angle. **(G)** Schematic drawing of interactions formed between DNL201 and the binding site. **(H)** Representative local densities with structural models of LRRK2^{RCKW}-DNL201 KIN, ROC, COR-A, COR-B and WD40 domains. All secondary structures of the KIN domain and one representative secondary structure of the rest domains are shown.

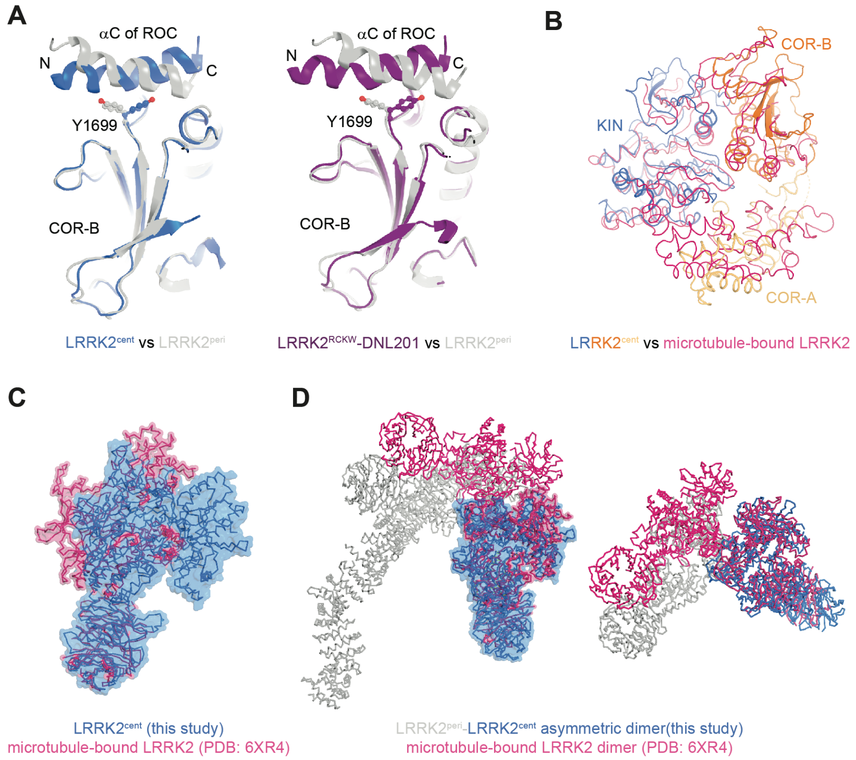

fig. S8: Comparison of different active conformations of LRRK2

(A) The seesaw motion of ROC αC helix between LRRK2^{cent} and LRRK^{peri} (left) and between LRRK2^{RCKW}-DNL201 and LRRK2^{peri} (right). LRRK2^{cent}, LRRK^{peri} and LRRK2^{RCKW}-DNL201 are colored in blue, grey and purple, respectively. **(B)** comparison of the KIN-COR interface between active LRRK2^{cent} and microtubule-bound LRRK2 (magenta) models (PDB: 6XR4). **(C)** Structural comparison of LRRK2^{cent} in the Rab29–LRRK2 tetramer and LRRK2 homodimer in the microtubule-bound LRRK2 structure. **(D)** Structural comparison between the asymmetric LRRK2 dimer in the Rab29–LRRK2 tetramer and the symmetric LRRK2 dimer in the microtubule-bound LRRK2 structure.

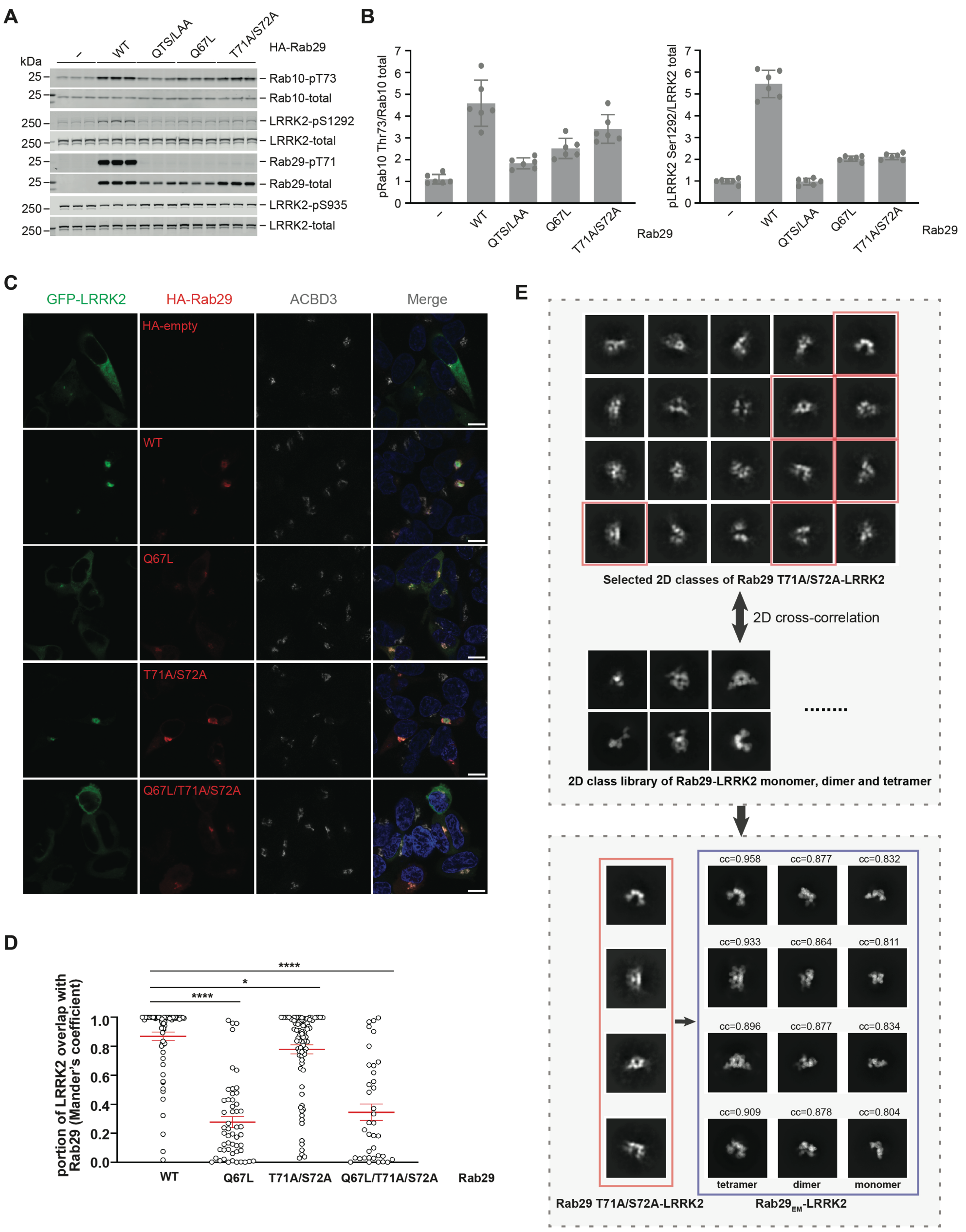

fig. S9: Structural and functional analysis of LRRK2 in the presence of wild-type or mutant Rab29.

(A) Quantitative immunoblotting analysis of the cellular kinase activity of wild-type LRRK2 in the presence of wild-type or mutant Rab29. HEK293 cells were transiently co-transfected with wild-type LRRK2 and either HA-empty vector (−) or the indicated variants of HA-tagged Rab29. Twenty-four hours post-transfection, cells were lysed and analyzed by quantitative immunoblotting using the indicated antibodies. QTS/LAA: Rab29$_{EM}$. **(B)** Quantification of the immunoblotting data shown in **A**. Data are presented as ratios of pRab10-Thr73/total Rab10 and pLRRK2-Ser1292/total LRRK2, normalized to the average of LRRK2 wild-type values. The data shown are the mean ± SD of three experiments. **(C)** Confocal analysis of LRRK2 recruitment to the Golgi by Rab29 wild-type or mutants. Wild-type GFP-LRRK2 (green) was co-expressed with wild-type or mutant (indicated) HA-Rab29 (red) in HEK293 cells, and localization was assessed

by confocal microscopy. The endogenous Golgi marker ACBD3 is also shown (white). Scale bar, 10 μm. **(D)** Quantification of Rab29 overlapping with LRRK2 by Mander's coefficient for confocal analysis shown in **(C)**. Error bars represent SEM of means. Significance was determined by the Kruskal-Wallis One-way ANOVA test. *p = 0.0286, ****p < 0.0001. **(E)** Validation of possible LRRK2 tetramer 2D classes from the Rab29 T71A/S72A–LRRK2 dataset by cross-correlation (cc) analysis. **Top:** cross-correlation analysis between 2D classes of Rab29 T71A/S72A–LRRK2 dataset and 2D classes from Rab29–LRRK2 tetramer, dimer and monomers. Red boxes indicate classes belong to Rab29–LRRK2 tetramer. Bottom: outcomes of cross-correlation analysis with scores indicated. Cc scores are between 0-1, with 1 being identical 2D images. All selected classes in the red box have the highest cc scores to the Rab29–LRRK2 tetramer state in the blue box, indicating Rab29 T71A/S72A–LRRK2 forms Rab29–LRRK2 tetramers. The tetramer ratio in Rab29 T71A/S72A–LRRK2 and $Rab29_{EM}$–LRRK2 are ~2% and 20%, respectively.

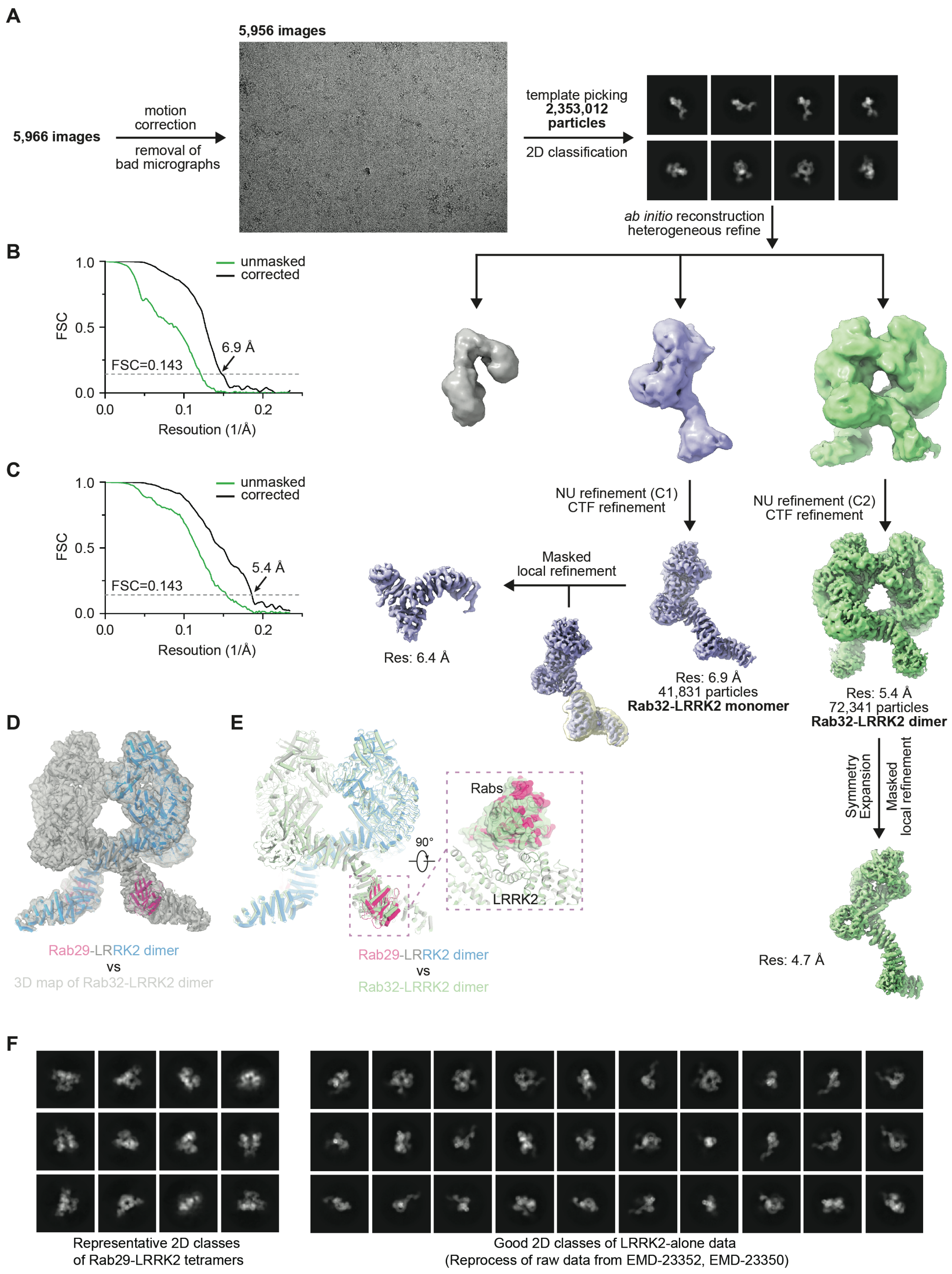

fig. S10: Structure of the Rab32–LRRK2 complex

(A) Structural determination of the Rab32–LRRK2 complex. **(B-C)** Fourier Shell Correlation (FSC) curves for the overall resolution of the Rab32–LRRK2 monomer and Rab32–LRRK2 dimer. **(D)** structural comparison between Rab32–LRRK2 and Rab29–LRRK2 complexes. The model of the Rab29–LRRK2 complex fits nicely into the Rab32–LRRK2 cryo-EM map. **(E)** Rab29 and Rab32 interact with LRRK2 on the same ARM repeats. **(F)** Reprocess of the LRRK2 alone data. **Left**: representative 2D classes of Rab29-LRRK2 tetramers. **Right**: all good classes of LRRK2-alone sample.

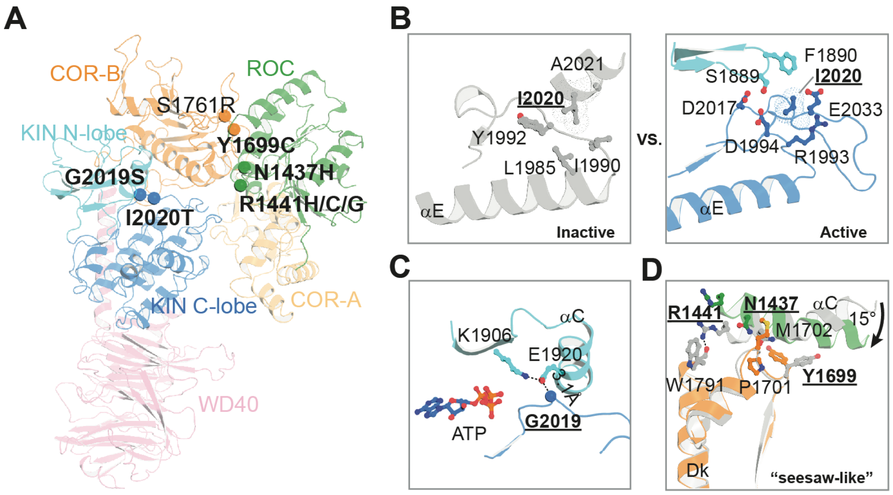

fig. S11: Disease mutations

(A) Locations of PD-causing mutation sites in the active state of LRRK2. The mutation sites are shown as spheres and labeled. **(B)** The chemical environment of Ile2020 in inactive and active LRRK2 molecules. **(C)** Position of the Gly2019 residue relative to the K1906-E1920 salt bridge in the active LRRK2. The residue Gly2019 is shown as a sphere, and the distance between Gly2019 and Glu1920 is labeled. **(D)** Tyr1699, Asn1437, and Arg1441 sites at the ROC-COR interface. The motion of the αC helix in the ROC domain between inactive and active states of LRRK2 is indicated by a black arrow.

Caption for Movie S1. Conformational changes of the catalytic half of LRRK2 from inactive to active states.

Caption for Movie S2. The "seesaw-like" motion of the αC helix of the ROC domain during an active-inactive-active conformational change cycle.

Table S1. Cryo-EM data collection, refinement, and validation statistics

	Rab29–LRRK2 monomer PDB 8FO2 EMD-29339	Rab29–LRRK2 dimer PDB 8FO8 EMD-29341	Rab29–LRRK2 tetramer PDB 8FO9 EMD-29342	$LRRK2^{RCKW}$–DNL201 PDB 8SMC EMD-40588
**Data collection and processing**				
Microscope/Camera		Titan krios/Gatan K3 Camera		
Magnification		81,000		130,000
Voltage (kV)		300		300
Total dose ($e^-/Å^2$)		58.77		68.81
Exp. rate (e^-/pixel/sec)		22.04		16.15
Frame number		60		60
Defocus range (mm)		0.6-1.8		1.0-2.8
Data collection software		EPU		EPU
Pixel size (Å)		1.06		0.6485
Total micrographs		11,762		33,456
Micrographs used		11,260		33,408
Symmetry imposed	C1	C2	C2	C1
Initial particle number	354,085	249,148	186,624	920,473
Final particle number	70,139	96,502	40,694	59,515
Map Resolution (global, Å)				
FSC-0.5: (unmasked/masked)	9.5/4.6	8.1/4.2	8.8/4.0	8.4/4.5
FSC-0.143: (unmasked/masked)	5.0/4.1	4.3/3.9	4.3/3.5	4.5/4.0
Resolution range (Å)	4.1-30.0	3.9-30.0	3.5-30.0	4.0-30.0
3DFSC sphericity value	0.781	0.985	0.975	0.525
**Refinement**				
Initial model used (PDB code)	7LHW and AlphaFold2	8FO2	8FO2 and AlphaFold2	8FO9
Refinement package	PHENIX	PHENIX	PHENIX	PHENIX
CC_{volume}/CC_{mask}	0.74/0.73	0.66/0.63	0.80/0.81	0.78/0.79
Model resolution (Å)	4.3	4.2	3.8	4.1
FSC threshold	0.5	0.5	0.5	0.5
Map sharpening *B* factor ($Å^2$)	-135.2	-120.0	-82.5	-118.1
Model composition				
Non-hydrogen atoms	18,231	36,334	53,769	7,674
Protein atoms	18,172	36,452	53,533	7,622
Ligand atoms	59	118	236	52
B factors ($Å^2$)				
Protein	61.12	40.55	111.61	41.02
Ligand	43.48	31.44	83.96	44.28
R.m.s deviations				
Bond lengths (Å)	0.004	0.005	0.004	0.003
Bond angles (°)	0.826	0.949	0.836	0.818

**Validation**				
MolProbity score	1.85	1.9	1.81	1.91
CaBLAM outliers (%)	4.4	4.4	3.5	4.6
EMRinger score	1.42	1.13	2.18	1.53
Clashscore	7.32	8.02	6.40	6.59
Poor rotamers (%)	0.00	0.00	0.11	0.00
Ramachandran plot				
Favored (%)	92.92	92.69	92.82	90.00
Allowed (%)	7.08	7.31	7.18	10.00
Disallowed (%)	0.00	0.00	0.00	0.00
